# The MIK2/SCOOP Signaling System Contributes to Arabidopsis Resistance Against Herbivory by Modulating Jasmonate and Indole Glucosinolate Biosynthesis

**DOI:** 10.3389/fpls.2022.852808

**Published:** 2022-03-23

**Authors:** Elia Stahl, Angel Fernandez Martin, Gaétan Glauser, Marie-Charlotte Guillou, Sébastien Aubourg, Jean-Pierre Renou, Philippe Reymond

**Affiliations:** ^1^Department of Plant Molecular Biology, University of Lausanne, Lausanne, Switzerland; ^2^Neuchâtel Platform of Analytical Chemistry, University of Neuchâtel, Neuchâtel, Switzerland; ^3^Institut de Recherche en Horticulture et Semences, UMR 1345, INRAE, Agrocampus-Ouest, Université d’Angers, Beaucouzé, France

**Keywords:** SCOOPs, phytocytokines, MIK2, plant-insect interactions, herbivory, glucosinolates, JA, JA-Ile

## Abstract

Initiation of plant immune signaling requires recognition of conserved molecular patterns from microbes and herbivores by plasma membrane-localized pattern recognition receptors. Additionally, plants produce and secrete numerous small peptide hormones, termed phytocytokines, which act as secondary danger signals to modulate immunity. In Arabidopsis, the *Brassicae*-specific SERINE RICH ENDOGENOUS PEPTIDE (SCOOP) family consists of 14 members that are perceived by the leucine-rich repeat receptor kinase MALE DISCOVERER 1-INTERACTING RECEPTOR LIKE KINASE 2 (MIK2). Recognition of SCOOP peptides elicits generic early signaling responses but knowledge on how and if SCOOPs modulate specific downstream immune defenses is limited. We report here that depletion of MIK2 or the single PROSCOOP12 precursor results in decreased Arabidopsis resistance against the generalist herbivore *Spodoptera littoralis* but not the specialist *Pieris brassicae.* Increased performance of *S. littoralis* on *mik2-1* and *proscoop12* is accompanied by a diminished accumulation of jasmonic acid, jasmonate-isoleucine and indolic glucosinolates. Additionally, we show transcriptional activation of the *PROSCOOP* gene family in response to insect herbivory. Our data therefore indicate that perception of endogenous SCOOP peptides by MIK2 modulates the jasmonate pathway and thereby contributes to enhanced defense against a generalist herbivore.

## HIGHLIGHTS

- The *PROSCOOP* gene family is activated in response to insect herbivory and SCOOP perception contributes to Arabidopsis resistance against *Spodoptera littoralis* by regulating jamsonate and indole glucosinolate biosynthesis.

## Introduction

In nature, plants are challenged by numerous biotic stressors throughout their life cycle and they have thus evolved sophisticated ways to respond to these attacks. Induction of robust plant immunity relies on pathogen and herbivore recognition. Plants activate immune signaling upon perception of non-self herbivore- and pathogen-associated molecular patterns (HAMPs, PAMPs) and self-derived damage-associated molecular patterns (DAMPs). Perception of those patterns is ensured by plasma membrane-localized pattern recognition receptors (PRRs) (Macho and Zipfel, 2014; Erb and Reymond, 2019; Ngou et al., 2021; Reymond, 2021). Additionally, plants are able to detect physical damage by wounding occurring during herbivore feeding. Upon perception of various HAMPs, PAMPs, and DAMPs, overlapping downstream signaling steps include membrane depolarization, a rapid Ca^2+^ influx, phosphorylation of the immune regulatory mitogen-activated protein kinases (MAPKs), production of reactive oxygen species (ROS), and transcriptional reprogramming (Fürstenberg-Hägg et al., 2013; Bigeard et al., 2015; Bjornson et al., 2021). Although early signaling events are commonly activated by plants in response to various pests, hormonal and metabolic responses are more distinct and specific to the nature of the invading organism. Plant immunity against biotrophic microbial pathogens is mainly controlled by salicylic acid (SA), whereas immunity against necrotrophic pathogens and herbivores is primarily orchestrated by jasmonic acid (JA) in its bioactive form jasmonate-isoleucine (JA-Ile) (Pieterse et al., 2012; Erb and Reymond, 2019; Peng et al., 2021). Efficient plant immunity requires moreover the endogenous production of numerous metabolites with direct antimicrobial and/or insecticidal activity (Stahl et al., 2018; Erb and Kliebenstein, 2020). One of the best-studied examples of insecticidal metabolites are glucosinolates that are found in plants of the order Brassicales. Aliphatic- and indole-glucosinolates (AGLs, IGLs) derive from methionine and tryptophan, respectively, and are constitutively produced as preformed defense compounds. Their biosynthesis is additionally inducible by various pest attacks, including insect infestation (Burow and Halkier, 2017). Upon tissue disruption, AGLs and IGLs are hydrolyzed by β-thioglucoside glucohydrolases to toxic aglycones, which can react spontaneously with biological nucleophiles and modify proteins and nucleic acids in the insect body (Pastorczyk and Bednarek, 2016). Inducible glucosinolate biosynthesis requires a functional JA signaling pathway and Arabidopsis mutants with disrupted JA signaling and glucosinolate biosynthesis or hydrolysis are highly susceptible to various arthropods, emphasizing the relevance of these pathways for robust immunity of cruciferous plants against herbivory (Barth and Jander, 2006; Beekwilder et al., 2008; Schweizer et al., 2013; Erb and Kliebenstein, 2020).

Plant generate and secrete numerous peptide hormones as signaling molecules which regulate growth, development and reproduction (Okuda, 2021). Additionally, several plant peptides act as immunogenic patterns. They serve as danger cell-to-cell signaling molecules to modulate immunity and are called phytocytokines (Luo, 2012; Gust et al., 2017; Yamaguchi and Kawasaki, 2021). HAMPs, PAMPs, DAMPs and phytocytokines are recognized by PRRs and largely overlap in early signaling events upon perception. However, how and if distinct phytocytokines modulate specific downstream immune responses is so far not well understood. The *Brassicae-specific* PRECURSOR OF SERINE-RICH ENDOGENOUS PEPTIDES (PROSCOOP) gene family consists of 14 homologs which encode for precursors of 14 SCOOP peptides in Arabidopsis (Gully et al., 2019). Various SCOOPs act as phytocytokines and activate immune responses in Arabidopsis, while SCOOP12 is suggested to function in regulating immunity but also in activating phospholipid signaling pathways and ROS production, thus controlling root development (Gully et al., 2019; Rhodes et al., 2021). The leucine-rich repeat receptor kinase (LRR-RK) MALE DISCOVERER 1-INTERACTING RECEPTOR-LIKE KINASE 2 (MIK2) was recently shown to be the common PRR for SCOOP peptides. SCOOP12 directly binds to the ectodomain of MIK2 and *mik2* mutant plants are insensitive to treatment with various synthetic SCOOP peptides. Moreover, perception of SCOOPs requires functional BRASSINOSTEROID INSENSITIVE 1-ASSOCIATED KINASE 1 (BAK1) and SCOOP12 causes a complex formation between MIK2 and the BAK1 co-receptor (Gully et al., 2019; Hou et al., 2021; Rhodes et al., 2021).

Phytocytokines are primarily described to function in immunity against microbial phytopathogens but knowledge on if and how phytocytokines modulate plant immunity against herbivorous insects is limited. In this study, we provide evidence that SCOOP peptide perception by MIK2 promotes herbivore-inducible IGL biosynthesis by modulating the JA signaling pathway and thereby contributes to Arabidopsis resistance against insect infestation. We moreover show enhanced transcription of the *PROSCOOP* gene family in response to herbivory and mechanical wounding, illustrating their role as phytocytokines whose perception by MIK2 can modulate plant immunity against herbivorous insects.

## Materials and Methods

### Plants, Insects, and Growth Conditions

*Arabidopsis thaliana* plants were vernalized for 2 days at 4°C and were cultivated in individual pots containing moist compost (Jiffy Substrates) in a controlled environmental growth chamber with a 10 h day/14 h night cycle. Experiments were conducted with 5-week-old plants. Mutant lines used in this study were described previously: *scoop12* CRISPR-Cas9-generated mutant in Col-0 background (Gully et al., 2019), *scoop12* T-DNA insertion line in Ws background (Gully et al., 2019) and *mik2-1* (Van der Does et al., 2017).

*Spodoptera littoralis* (Egyptian cotton worm) eggs were obtained from Syngenta (Stein AG; Switzerland). For hatching, *S. littoralis* eggs were incubated for 48 h at 28°C. *Pieris brassicae* (Large White butterfly) was reared in a greenhouse on *Brassica oleracea* var. *gemmifera* as described previously (Bonnet et al., 2017).

### Insect Performance, Herbivory and Mechanical Wounding

For measurements of insect performance, 15–20 freshly hatched *P. brassicae* or 40–65 freshly hatched *S. littoralis* larvae were placed on 11 plants per genotype in transparent plexiglass boxes. *P. brassicae* and *S. littoralis* larvae were allowed to feed on those plants for 10 and 12 days respectively and individual larval weights were determined subsequently on a high precision balance (Mettler-Toledo; XP205DR, Switzerland).

Samples for JA, glucosinolate and gene expression analysis were taken after 2 days of *S. littoralis* feeding. Eight to ten uniformly infested fully developed leaves from 8 to 10 individual plants were harvested per sample and experiment. Eight to ten healthy leaves from 8 to 10 non-infested plants served as controls. The full samples were homogenized and aliquots were used for JA, RNA and glucosinolate extractions.

For mechanical wounding, 15 leaves of three plants (five leaves per plant) were wounded by cutting four holes (1 mm radius) per leave. Wounded leaves were harvested 4 and 24 h post wounding. Fifteen healthy non-wounded leaves from three plants served as controls. The 15 leaves were pooled to one sample per experiment, homogenized and aliquots were used for RNA extractions.

### SCOOP12 Treatment

The SCOOP12 peptide (PVRSSQSSQAGGR) was synthesized by Eurogentec SA (Angers, France) and diluted in distilled Milli-Q H_2_O to final concentrations used for the experiments. Twelve uniformly developed healthy leaves out of six individual plants were infiltrated with 1 μM SCOOP12 per experiment, using a 1 ml needleless syringe. Twelve leaves out of six different individual plants, infiltrated with distilled H_2_O, served as controls. Samples were taken 24 h after the infiltration. The 12 leaves were pooled to one sample per experiment, homogenized and aliquots were used for glucosinolate and RNA extractions.

### Measurement of Jasmonic Acid and Jasmonate-Isoleucine

Analysis of JA and JA-Ile was performed using a protocol adapted from Glauser et al. (2014). Briefly, approximately 100 mg of leaf material was extracted in 990 ul of ethylacetate:formic acid (99.5:0.5, v/v) and 10 μl of an internal standard solution containing JA-d_5_ and JA-Ile-^13^C_6_ at 100 ng/ml. After centrifugation, the pellet was re-extracted with 0.5 ml of ethylacetate:formic acid (99.5:0.5, v/v) and both supernatants were combined and evaporated at 35°C. The dried residue was reconstituted in 0.2 ml of methanol 50% and 2 μl were injected in a UHPLC-MS/MS system composed of an Acquity UPLC (Waters) and a QTRAP 6500+ (Sciex). The final concentration of internal standards was 5 ng/ml.

### Glucosinolate Analysis

Determination of aliphatic and indole glucosinolates was performed as described previously by Glauser et al. (2012) with minor modifications. Briefly, approximately 50 mg of homogenized leaf material was weighted and suspended in 1 ml ice-cold methanol:water:formic acid (70:30:0.1) by vortexing. Five small glass beads where added per sample and samples were shaken for 3 min at 30 Hz in a Qiagen TissueLyser II bead mill. Samples were centrifuged for 3 min at 14,000 × *g* and 200 μl of the supernatant was transferred to a new tube. Subsequently, a small aliquot was dissolved 10-fold with the extraction solvent and injected into an Acquity UPLC I-class coupled to a Synapt XS QTOF (Waters) for absolute quantification of glucosinolates as described previously (Glauser et al., 2012). Glucosinolate levels are given in μg g^–1^ fresh weight.

### Gene Expression Analysis

Analysis of gene expression was conducted as described previously (Stahl et al., 2020). In short, total RNA extraction was performed by the use of the Relia Prep RNA Tissue Mini Prep System (Promega). For reverse transcription by M-MLV reverse transcriptase 1 μg of total RNA was used. cDNA synthesis was conducted in triplicates and obtained cDNA was diluted eightfold with water for subsequent quantitative real-time PCR (qPCR) analysis. qPCR analysis was performed in a total volume of 20 μl containing 10 mL of Brilliant III Ultra Fast SYBR Green QPCR Master Mix (Agilent), 0.2 μM of each primer, 0.03 μM of reference dye (ROX) and 2 μl of cDNA on a QuantStudio three real-time PCR machine (Applied Biosystems; Thermo Scientific) with the following temperature program: 95°C for 3 min, then 40 cycles of 10 s at 95°C and 20 s at 60°C. Primers for qPCR analysis used in this study are given in Supplementary Table S6.

### Reactive Oxygen Species Measurement

Leaf disks (4 mm-diameter) were harvested from 4-week-old plants. Two leaf disks from six individual plants per genotype and treatment were used for the analysis and were floated overnight in 100 μl distilled H_2_O in a white 96-well plate (Thermo Scientific). For ROS assay, the water was removed and replaced with 100 μl assay solution, containing 10 μg ml^–1^ Pierce™ horseradish peroxidase (Thermo Scientific) and 100 μM of L-012 (Merck). Luminescence was measured immediately after the addition of 1 μM SCOOP12 for 60 min (1 measurement per minute) on a HIDEX Sense microplate reader with an integration time of 0.1 s. Leaf disks treated with distilled H_2_O served as controls.

### Reproducibility of Experiments and Statistical Analyses

All results presented in this study represent the mean ± SEM of three independent biological experiments, except the ROS measurements for verification of SCOOP12 activity and non-responsiveness of *mik2-1* (Supplementary Figure S5A), which were conducted once with six individual plants per genotype and treatment. Different biological experiments are indicated with different symbol shapes (circle, square and triangle) in the corresponding figures. Normal distribution of the data was determined by Shapiro–Wilk test. Statistical differences for pairwise comparisons for insect bioassays were evaluated by Mann–Whitney *U* test. Statistical differences between *S. littoralis*-inducible transcript levels were determined by a ratio paired *t*-test, pairing different biological replicates. Multiple comparisons between glucosinolate and JA levels were performed by analysis of variance (ANOVA) followed by Tukey’s HSD *post-hoc* test. The choice of statistical analysis is given in the corresponding figure/table legend.

## Results

### MIK2 Is Involved in Arabidopsis Resistance Against Herbivorous Insects

**FIGURE 1:**
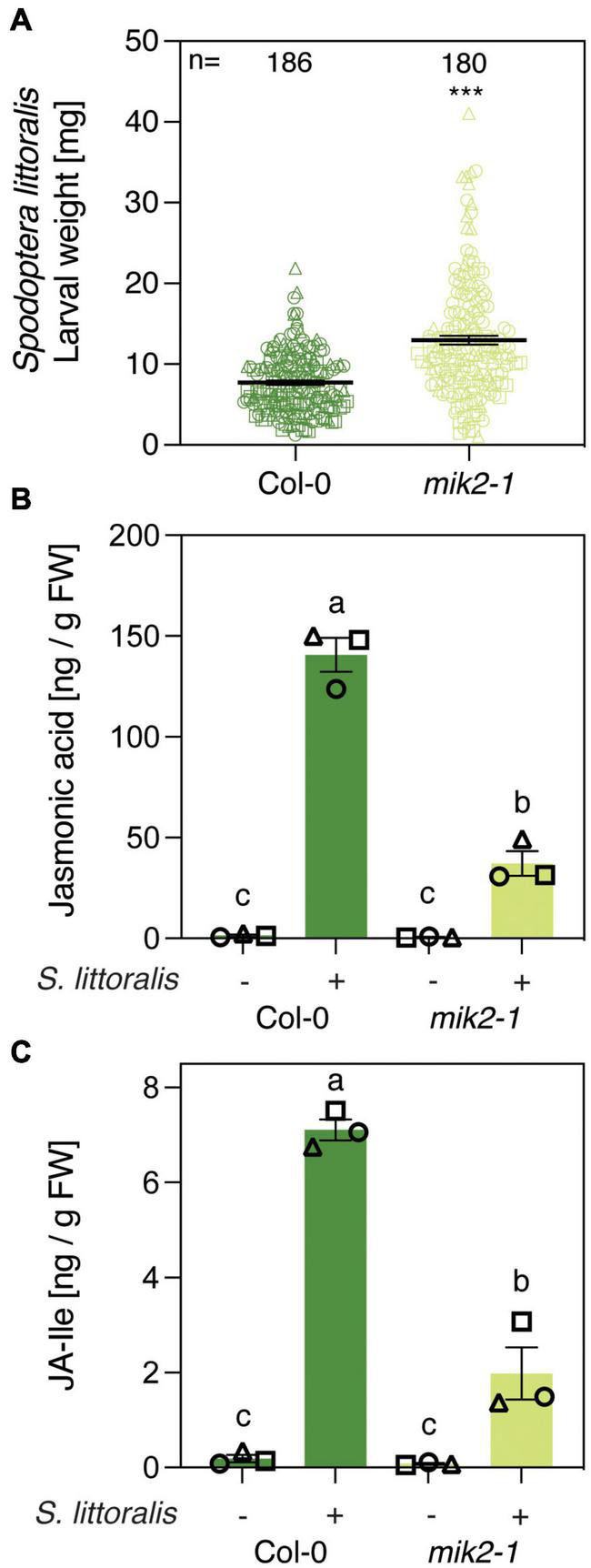
MIK2 contributes to Arabidopsis immunity against a generalist herbivore. **(A)** Insect performance of *Spodoptera littoralis* on Col-0 and *mik2-1*. *S. littoralis* larvae were feeding on 5-week-old plants for 12 days. Means ± SEM of three independent biological replicates are shown. Asterisks denote statistical differences between larvae feeding Col-0 and *mik2-1*: ***P < 0.001 (Mann–Whitney *U* test). Symbols indicate individual values and symbol shapes (circle, square, and triangle) indicate different biological replicates. **(B,C)** Jasmonate levels in Col-0 and *mik2-1*. Absolute levels of total JA **(B)** and JA-Ile **(C)** were measured by UHPLC-MS/MS after 2 days of *S. littoralis* feeding. Non-infested plants served as controls. Values represent means ± SEM of three independent biological replicates. Letters denote statistical differences (ANOVA followed by Tukey’s HSD). Different symbols indicate different biological replicates.

Transcriptional profiling of roots of Arabidopsis seedlings revealed an upregulation of genes involved in plant immunity and indole glucosinolate biosynthesis upon SCOOP12 perception (Guillou et al., 2021). MIK2 is the common receptor for SCOOP peptides in Arabidopsis and *mik2* mutants are insensitive to various SCOOP peptides (Hou et al., 2021; Rhodes et al., 2021). We therefore tested if knocking out MIK2 results in amended plant-responses to herbivorous insects. Five-week-old *mik2-1* plants were infested with freshly hatched larvae of the generalist *Spodoptera littoralis* and the specialist *Pieris brassicae* for 12 and 10 days, respectively, and larval weight was determined subsequently to measure insect performance. Interestingly, larvae of the generalist *S. littoralis* gained significantly more weight on *mik2-1* compared to the Col-0 wild-type control (Figure 1A). These results were supported by more consumed leaf material of *mik2-1* during the bioassay and an increased average size of *S. littoralis* larvae (Supplementary Figure S1A). By contrast, *P. brassicae* larvae were significantly smaller when feeding on *mik2-1* (Supplementary Figure S1B). Plant defense against herbivorous insects is primarily regulated by JA in its bioactive form JA-Ile (Howe et al., 2018). We therefore measured the accumulation of JA and JA-Ile upon *S. littoralis* infestation in Col-0 and *mik2-1* (Figures 1B,C). In accordance with increased *S. littoralis* performance on *mik2-1*, *S. littoralis*-inducible levels of JA and JA-Ile were significantly diminished in *mik2-1* compared to Col-0, implying a functional role for MIK2 in modulating the JA pathway in response to herbivorous arthropods.

**FIGURE 2:**
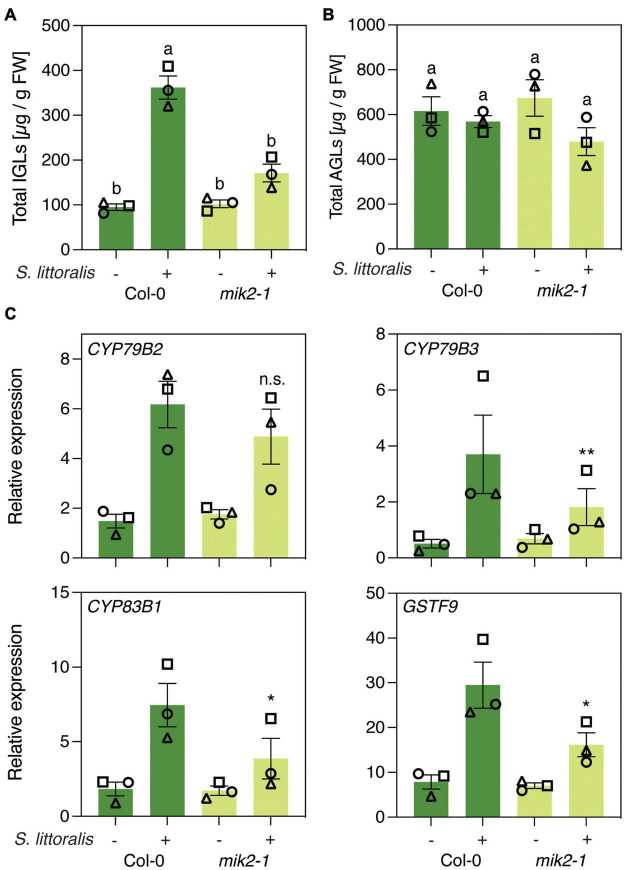
MIK2 regulates indole glucosinolate biosynthesis in response to herbivory. **(A,B)** Glucosinolate levels in Col-0 and *mik2-1*. Absolute levels of total IGLs **(A)** and AGLs **(B)** were measured by UPLC-QTOF after 2 days of *Spodoptera littoralis* feeding. Non-infested plants served as controls. Values represent means ± SEM of three independent biological replicates. Letters denote statistical differences (ANOVA followed by Tukey’s HSD). Different symbols indicate different biological replicates. Values for individual glucosinolate species are given in Supplementary Table S1. **(C)** Expression of genes involved in indole glucosinolate biosynthesis. Expression of *CYP79B2*, *CYP79B3*, *CYP83B1*, and *GSTF9* was measured by qPCR after 2 days of *Spodoptera littoralis* feeding and normalized to the housekeeping gene *SAND*. Non-infested plants served as controls. Values represent means ± SEM of three independent biological experiments. Asterisks denote statistical differences between *S. littoralis-*induced expression levels of Col-0 and *mik2-1*: *P < 0.05, **P < 0.01, n.s., no significant difference (ratio paired *t*-test). Different symbols indicate different biological replicates.

Then, to investigate if increased *S. littoralis* performance on *mik2-1* is due to altered glucosinolate levels, we measured levels of IGLs and AGLs in response to *S. littoralis* infestation. Levels of IGLs increased in response to *S. littoralis* feeding in Col-0 and this accumulation was significantly reduced in *mik2-1* (Figure 2A and Supplementary Table S1). However, constitutive IGL levels were not affected in *mik2-1*, indicating that SCOOP perception could promote *S. littoralis* inducible IGLs but is not implicated in basal IGL accumulation. The total amount of AGLs did not change upon *S. littoralis* feeding, irrespective of the genotype (Figure 2B and Supplementary Table S1). Of note, the AGL 7-methylthioheptyl-glucosinolate (7MTH) accumulated significantly in response to *S. littoralis* infestation in Col-0 but not in *mik2-1* (Supplementary Table S1).

We next measured if genes involved in IGL biosynthesis were differentially regulated in *mik2-1* upon herbivory. The two cytochrome P450 monooxygenases CYP79B2 and CYP79B3 catalyze an initial step in IGL biosynthesis by converting Trp into indole-3-acetaldoxime (IAOx), which serves as a precursor for several indole-derived metabolites (Zhao et al., 2002). The cytochrome P450 monooxygenases CYP83B1 and the glutathione-S-transferase 9 (GSTF9) are involved in metabolizing IAOx to glucobrassicin (I3M), which constitutes the main IGL in Arabidopsis (Sønderby et al., 2010; Supplementary Tables S1–S3). *CYP79B2*, *CYP79B3*, *CYP83B1* and *GSTF9* transcript levels increased in response to *S. littoralis* infestation in Col and *mik2-1* (Figure 2C). However, *S. littoralis*-induced *CYP79B3*, *CYP83B1* and *GSTF9* were significantly reduced in *mik2-1* compared to Col-0, indicating a regulatory role for SCOOP peptide perception in herbivore-inducible IGL biosynthesis. Similar transcriptional patterns were observed for the basic helix-loop-helix transcription factor *MYC2*, the *VEGETATIVE STORAGE PROTEIN 2* (*VSP2*) and the *JASMONATE ZIM DOMAIN PROTEINS 5* and *10* (*JAZ5*, *JAZ10*), all of which constitute a hallmark of induced immunity against herbivorous insects. Consistent with decreased *S. littoralis*-inducible JA levels in *mik2-1*, this data suggests that MIK2 modulates plant immunity against herbivorous insects via the JA pathway, of which IGL biosynthesis constitutes one of the downstream elements. However, a significant difference for gene expression was only observed for *MYC2* (Supplementary Figure S2).

### *PROSCOOP12* Contributes to Indole Glucosinolate Biosynthesis in Response to Herbivory

**FIGURE 3:**
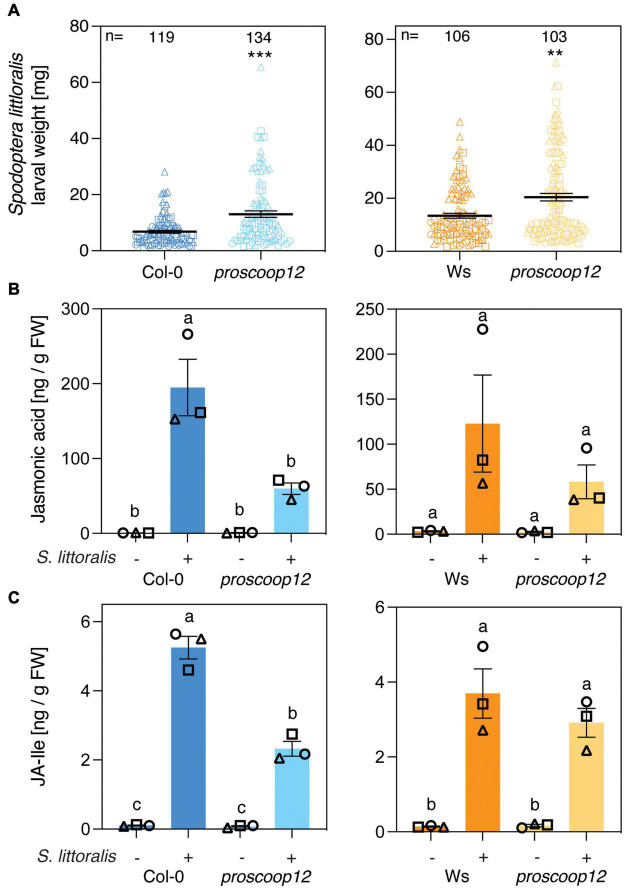
*PROSCOOP12* modulates Arabidopsis defense against *Spodoptera littoralis*. **(A)** Insect performance of *S. littoralis* on *proscoop12* mutants in Col-0 and Ws backgrounds. *S. littoralis* larvae were feeding on 5-week-old plants for 12 days. Means ± SEM of three independent biological replicates are shown. Asterisks denote statistical differences between mutant plants and wild-type controls: **P < 0.01, ***P < 0.001 (Mann–Whitney *U* test). Symbols indicate individual values and symbol shapes (circle, square, and triangle) indicate different biological replicates. **(B,C)** Jasmonate levels in *proscoop12* mutants and the corresponding wild-type controls. Absolute levels of total JA **(B)** and JA-Ile **(C)** were measured by UHPLC-MS/MS after 2 days of *S. littoralis* feeding. Non-infested plants served as controls. Values represent means ± SEM of three independent biological replicates. Letters denote statistical differences (ANOVA followed by Tukey’s HSD). Different symbols indicate different biological replicates.

The involvement of MIK2 in Arabidopsis immunity upon herbivory led to the question if depletion of a single SCOOP peptide affects activation of plant immune signaling in response to herbivore infestation. Due to upregulation of genes involved in plant immunity and indole glucosinolate biosynthesis in response to SCOOP12 perception (Guillou et al., 2021), we tested if mutations in the precursor gene *PROSCOOP12* result in attenuated plant immunity against herbivorous insects. The experiments were conducted with two independent knock-out mutants, a CRISPR-Cas9 line in the Col-0 background and a T-DNA insertion line in the Wassilewskija (Ws) background (Gully et al., 2019). We first measured *S. littoralis* performance on the two *proscoop12* mutants and the corresponding wild-type controls. *S. littoralis* gained significantly more weight on *proscoop12* compared to the corresponding wild-type control, consistent with the effect observed for *mik2-1* (Figure 3A). Larvae of the specialist *P. brassicae* gained the same weight irrespective of the genotype they were allowed to feed on (Supplementary Figure S3). JA and JA-Ile accumulated in Col-0, Ws and the two *proscoop12* mutants in response to *S. littoralis* attack (Figures 3B,C). In line with data obtained with *mik2-1*, herbivore-inducible JA and JA-Ile levels were reduced in *proscoop12*, although significant differences could just be observed in the Col-0 background.

**FIGURE 4:**
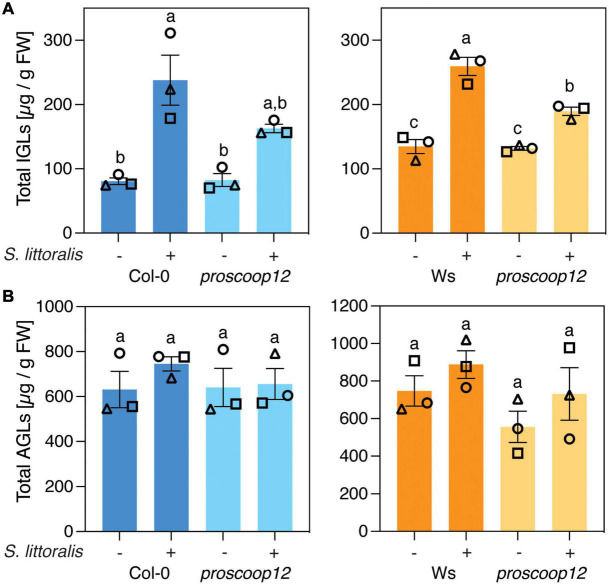
*PROSCOOP12* modulates indole glucosinolate biosynthesis in response to herbivory. **(A,B)** Glucosinolate levels in *proscoop12* mutants and corresponding wild-type controls. Absolute levels of total IGLs **(A)** and AGLs **(B)** were measured by UPLC-QTOF after 2 days of *S. littoralis* feeding. Non-infested plants served as controls. Values represent means ± SEM of three independent biological replicates. Letters denote statistical differences (ANOVA followed by Tukey’s HSD). Values for individual glucosinolate species are given in Supplementary Table S2. Different symbols indicate different biological replicates.

Then, we measured the activation of IGL biosynthesis in *proscoop12* and the corresponding wild-type controls. Strikingly, levels of IGLs increased upon *S. littoralis* infestation in Col-0 and Ws but this accumulation was less pronounced in *proscoop12* mutants (Figure 4A and Supplementary Table S2), consistent with reduced JA and JA-Ile levels in the same lines (Figures 3B,C). Again, the total amount of AGLs did not change upon *S. littoralis* feeding, irrespective of the genotype (Figure 4B and Supplementary Table S2).

**FIGURE 5:**
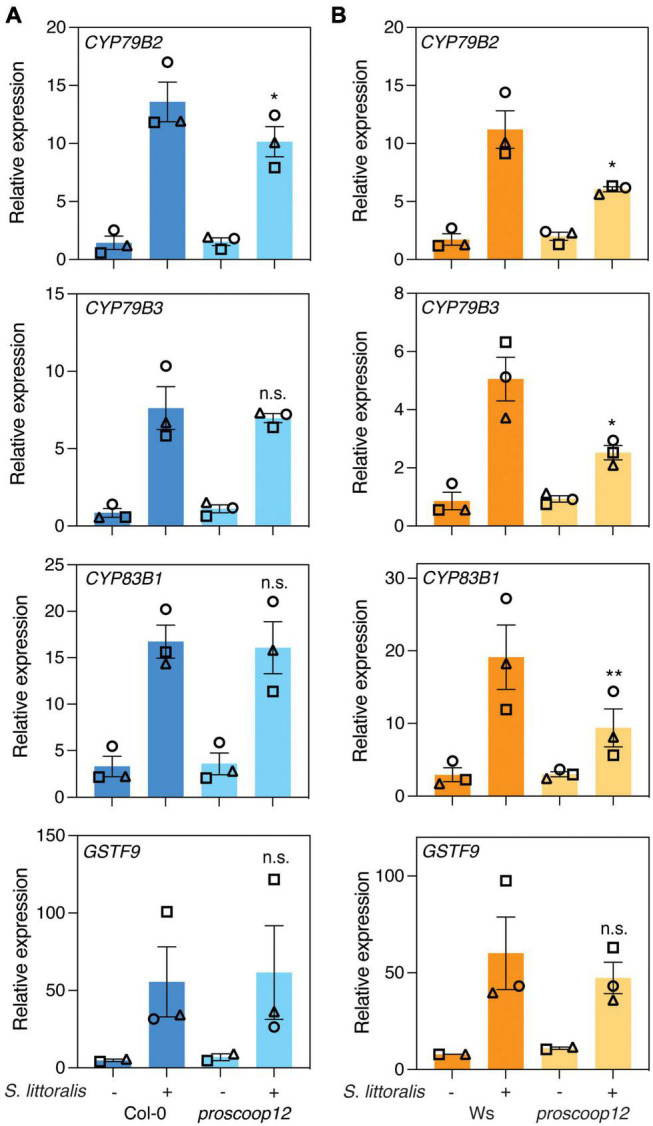
Expression of genes involved in indole glucosinolate biosynthesis. Expression of *CYP79B2*, *CYP79B3*, *CYP83B1*, and *GSTF9* was measured by qPCR after 2 days of *Spodoptera littoralis* feeding on *proscoop12* mutants in Col-0 **(A)** and Ws **(B)** backgrounds. Expression was normalized to the housekeeping gene *SAND*. Non-infested plants served as controls. Values represent means ± SEM of three independent biological replicates. Asterisks denote statistical differences between *S. littoralis-*induced expression levels in *proscoop12* and the corresponding wild-type control: *P < 0.05, **P < 0.01, n.s., no significant difference (ratio paired *t*-test). Different symbols indicate different biological replicates.

We next examined the expression of *CYP79B2*, *CYP79B3*, *CYP83B1* and *GSTF9* upon *S. littoralis* infestation in Col-0, Ws and *proscoop12* mutant lines (Figure 5). All four genes were induced in Col-0 and Ws in response to *S. littoralis* infestation and there was a general trend for lower induction in the *proscoop12* mutant (Ws background). However, a significant difference was only observed for *CYP79B2*, *CYP79B3* and *CYP83B1*. Also, these genes were equally induced in *proscoop12* and wild-type in the Col-0 background with the exception of *CYP97B2* which showed a minor but significant reduced induction in *proscoop12* (Col-0 background). Similar tendencies were observed for *MYC2*, *VSP2*, *JAZ5*, and *JAZ10* (Supplementary Figure S4), supporting the hypothesis that by activating MIK2, SCOOP peptides indirectly contribute to plant defense against herbivorous insects by modulating the JA pathway.

The attenuated plant defense against *S. littoralis* in *proscoop12* and *mik2-1* mutants led to the question if activation of a SCOOP/MIK2 complex could regulate defense signaling. We therefore tested if exogenous application of SCOOP12 is sufficient to activate the above described responses. We first verified SCOOP12 activity and SCOOP-insensitivity of *mik2-1* by the ability of the peptide to activate apoplastic ROS production (Supplementary Figure S5A; Rhodes et al., 2021). Next, we infiltrated leaves of Col-0 and *mik2-1* with 1 μM of SCOOP12 and measured accumulation of IGLs, AGLs and transcript levels of *CYP79B2*, *CYP79B3* and *MYC2* 24 h later. Although SCOOP12 treatment is sufficient to activate the rapid and transient production of extracellular ROS in a MIK2-dependent manner (Supplementary Figure S5A; Hou et al., 2021; Rhodes et al., 2021; Gully et al., 2019), infiltration of SCOOP12 did not lead to increased levels of glucosinolates (Supplementary Figures S5B,C and Supplementary Table S3) or to increased expression of *CYP79B2*, *CYP79B3* and *MYC2* (Supplementary Figure S5D). Therefore, this indicates that perception of SCOOP12 alone is not sufficient to activate plant defense against herbivorous insects and implies a regulatory role for SCOOP12 perception downstream of the initial recognition of herbivore attack to enhance plant immunity.

### Various *PROSCOOPs* Are Induced Upon Herbivory and Mechanical Wounding

**FIGURE 6:**
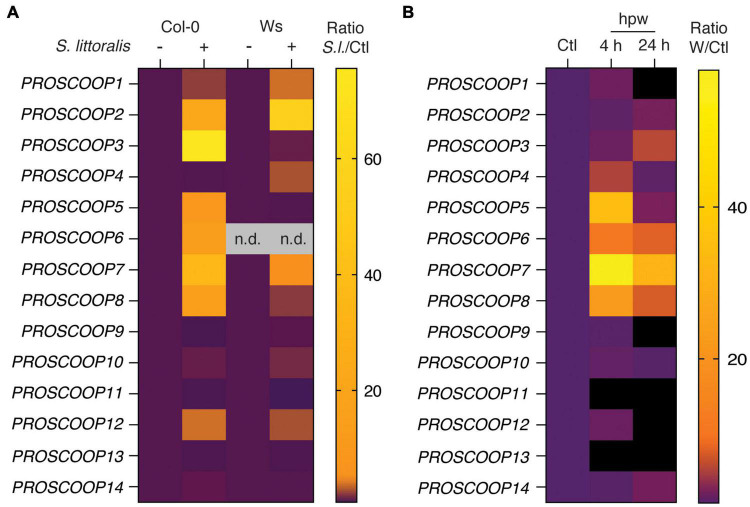
Heatmap of *PROSCOOP* expression in response to *Spodoptera littoralis* feeding and mechanical wounding. **(A)** Expression of *PROSCOOP1* to *PROSCOOP14* in Col-0 and Ws after 2 days of *S. littoralis* feeding. Fold changes are normalized to the corresponding non-infested controls and represent the means of three independent experiments. n.d., not detectable. Expression levels relative to the housekeeping gene *SAND* are given in Supplementary Table S4. **(B)** Expression of *PROSCOOP1* to *PROSCOOP14* in response to mechanical wounding. Expression levels were determined 4 and 24 h after mechanical wounding, are normalized to the levels of non-wounded plants and represent the mean of three independent experiments. hpw, hours post wounding. Expression levels relative to the housekeeping gene *SAND* are given in Supplementary Table S5.

The SCOOP family consists of 14 different SCOOP peptides in Arabidopsis for which several members are suggested to partially overlap in their functionality as immunogenic patterns (Gully et al., 2019; Rhodes et al., 2021). We thus measured the transcript levels of genes for the precursors *PROSCOOP1* to *PROSCOOP14* upon *S. littoralis* infestation by qPCR (Figure 6A and Supplementary Table S4). Transcript levels of various *PROSCOOPs*, including *PROSCOOP1*, *2*, *3*, *4*, *5*, *6*, *7*, *8* and *12*, were induced in Col-0 and/or Ws, suggesting a generic involvement of SCOOP peptides in Arabidopsis immunity against herbivorous insects. Notably, *PROSCOOP6* transcripts were not detectable in Ws, which is consistent with a lack of *PROSCOOP6* reads in RNAseq experiments in Ws and illustrates the natural variation in *PROSCOOP* duplicated genes between Arabidopsis ecotypes (Guillou et al., 2021).

Beside the recognition of HAMPs, wounding is an important component of plant responses to chewing herbivores (Stahl et al., 2018). Therefore, we next determined transcript levels of the 14 *PROSCOOPs* in response to mechanical wounding (Figure 6B; Supplementary Table S5). Consistent with *PROSCOOP* induction upon *S. littoralis* feeding, transcript levels of *PROSCOOP4*, *5*, *6*, *7* and *8* accumulated in Col-0 4 h post wounding and similar patterns could be observed 24 h post wounding although the response was less pronounced.

## Discussion

Plants activate immune signaling upon recognition of various self and non-self molecules (Gust et al., 2017; Yamaguchi and Kawasaki, 2021). PAMPs and HAMPs are involved in initial pathogen and herbivore recognition, respectively, whereas phytocytokines are secreted by plants to modulate immunity and thereby act as secondary danger signals. Primary and secondary signals are perceived by PRRs and share common early signaling events upon perception (Gust et al., 2017). Previous studies have reported crucial roles for several phytocytokines, such as plant elicitor peptides (PEPs), PAMP-induced peptides (PIPs) and systemin, as they reinforce plant immunity against various pests (Huffaker et al., 2006, 2011, 2013; Hou et al., 2014; Klauser et al., 2015; Shinya et al., 2018; Wang et al., 2018; Xu et al., 2018; Poretsky et al., 2020). We show here enhanced expression of the *PROSCOOP* gene family in response to herbivory in two Arabidopsis accessions, indicating a role for these phytocytokines in Arabidopsis resistance against chewing herbivores. Additionally, genes coding for several PROSCOOPs were also induced in wounded plants, highlighting wounding as an important component of herbivory recognition and confirming partial overlapping transcriptional changes upon insect infestation and mechanical wounding in Arabidopsis (Reymond et al., 2000). Depletion of the generic SCOOP receptor MIK2 or the single PROSCOOP12 precursor led to increased performance of the generalist *S. littoralis*, emphasizing a potential role for SCOOP peptide synthesis and perception in Arabidopsis resistance against herbivorous insects. Given that differences in larval performance were more pronounced on *mik2-1*, the SCOOP peptide family might have additive effects on plant resistance against chewing insects. These observations are in line with recent reports, which suggest that the SCOOP peptide family shares MIK2 as a common receptor in Arabidopsis and overlap in their functionality as immunogenic patterns (Hou et al., 2021; Rhodes et al., 2021). Notably, *PROSCOOP12* transcript levels just moderately increased in response to *S. littoralis* infestation. Therefore, an interesting question for further investigations is if the depletion of highly induced PROSCOOP precursors, such as *PROSCOOP2*, *3* and *7*, leads to attenuated immunity against herbivorous insects in Arabidopsis. Active plant peptides are derived from precursor proteins by proteolytic cleavage (Hander et al., 2019; Chen et al., 2020). However, proteases processing PROSCOOP precursors to generate SCOOP peptides have not been identified yet. Hence, characterization of these proteases and processing of PROSCOOPs in response to herbivory are important aspects which deserve further investigations.

Interestingly, increased performance of *S. littoralis* on *mik2-1* and *proscoop12* was accompanied by a diminished accumulation of JA and JA-Ile, which constitute the primary mediators of plant immunity against insect herbivores (Howe et al., 2018). A similar trend was found for the JA-signaling genes *MYC2*, *JAZ5* and *JAZ10*, and the JA-responsive marker *VSP2*. Collectively, this suggests that SCOOP peptide perception by MIK2 modulates Arabidopsis immunity against chewing herbivores by targeting the JA pathway. However, the precise molecular mechanism that connects SCOOP perception to JA signaling is currently unknown and will require further investigation. One of the best characterized JA-dependent defense response against herbivorous insects in Arabidopsis is the biosynthesis of glucosinolates. Arabidopsis resistance against chewing herbivores highly relies on glucosinolates, which are produced and stored in Arabidopsis constitutively and act as phytoanticipins in basal immunity. Their synthesis is also induced in response to various pathogen attacks and insect infestation and their breakdown products exert direct insecticidal activity (Barth and Jander, 2006; Beekwilder et al., 2008; Schweizer et al., 2013; Pastorczyk and Bednarek, 2016; Erb and Kliebenstein, 2020). Interestingly, *S. littoralis*-inducible but not basal IGL levels were lower in *mik2-1* and *proscoop12* compared to the corresponding controls, indicating a regulatory role for SCOOP peptide perception in herbivore-inducible IGL biosynthesis. This hypothesis is strengthened by recent findings, which show an upregulation of genes involved in IGL biosynthesis in response to SCOOP12 treatment in roots of Arabidopsis seedlings (Guillou et al., 2021). Therefore, the lower *S. littoralis*-inducible IGL levels in *mik2-1* and *proscoop12* likely explain the increased *S. littoralis* performance on these lines. As for larval performance and jasmonate levels, the differences in herbivore-inducible IGL levels were more pronounced in *mik2-1* compared to *proscoop12*, suggesting again additive effects of diverse SCOOP peptides on Arabidopsis resistance against this generalist herbivore. By contrast, performance of the specialist *P. brassicae* was either reduced (in *mik2-1*) or not affected (in *proscoop12*) by altered IGL levels and this is presumably due to its ability to detoxify glucosinolates (Schlaeppi et al., 2008; Schweizer et al., 2013). Indeed, *P. brassicae* recognizes appropriate host plants by detecting the presence of glucosinolates (Schweizer et al., 2013). Therefore, the lower performance of *P. brassicae* on *mik2-1* might be explained by less feeding stimulants due to lower IGL levels, a phenomenon described previously (Barth and Jander, 2006). Further studies with adapted and non-adapted herbivores will be needed to confirm the specific role of MIK2 and SCOOP peptides in IGL-dependent resistance.

Activation of indole metabolism in Arabidopsis is not limited to herbivore-infested plants. For instance, accumulation of IGLs and other indolics was reported in response to various abiotic and biotic stressors such as ROS, PAMPs, microbial pathogens and insect egg recognition (Sewelam et al., 2014; Frerigmann, 2016; Frerigmann et al., 2016; Stahl et al., 2016; Alfonso et al., 2021). Genes coding for several PROSCOOPs are induced in response to *Botrytis cinerea* and *Pseudomonas syringae* in Arabidopsis and it will be therefore an interesting aspect for further investigations to study if and how SCOOP peptides contribute to the synthesis of IGLs and other indolics in these conditions (Gully et al., 2019). We measured here the expression of *CYP79B2*, *CYP79B3*, *CYP83B1* and *GSTF9*, which all catalyze crucial steps in IGL biosynthesis. All four genes were induced in the wild-type controls, *mik2-1* and *proscoop12* after *S. littoralis* feeding. Nonetheless, this induction was often lower in *mik2-1* and in some cases moderately decreased in *prosccop12* and we assume that this explains the strongly decreased *S. littoralis*-inducible IGL levels observed in *mik2-1* and the slightly decreased ones in *proscoop12*. Thereby, SCOOP peptides might modulate IGL biosynthesis moderately and subtle decreased expression at each biosynthetic step may result in overall decreased IGL levels. However, expression analysis of selected genes at one specific timepoint just reveals a snapshot of the complex transcriptional reprogramming following herbivore attack. Therefore, we cannot rule out the possibility that SCOOP perception mediates another crucial step in IGL biosynthesis than the induction of the above-mentioned genes. An alternative explanation for reduced herbivore-inducible IGL levels in *mik2-1* and *procoop12* is a potential involvement of SCOOPs in modulating post-transcriptional regulation of IGL biosynthesis. For instance, protein phosphatase 2A-dependent dephosphorylation of enzymes involved in IGL biosynthesis has been reported previously to be a crucial component of IGL biosynthesis in Arabidopsis (Rahikainen et al., 2017).

We additionally tested if exogenous application of SCOOP12 elicits an activation of IGL biosynthesis. However, infiltration of SCOOP12 did not lead to an increased accumulation of IGLs, expression of genes involved in IGL biosynthesis or expression of *MYC2*, suggesting that SCOOP12 is not an elicitor of these responses. Natural wounding by chewing insects is a complex situation involving the recognition of numerous of HAMPs and DAMPs, hydrostatic pressure changes, secretion and perception of phytocytokines and is altered by herbivore-derived effectors (Consales et al., 2012; Farmer et al., 2014; Gust et al., 2017; Stahl et al., 2018; Erb and Reymond, 2019; Snoeck et al., 2022). Based on this complexity, we believe that it is unlikely that one pattern alone is sufficient to trigger the signaling cascade leading to the activation of IGL biosynthesis and suggest a scenario in which SCOOP peptide perception by MIK2 could boost the JA pathway downstream of initial herbivore recognition for robust plant immunity. However, we cannot formally exclude the possibility that exogenous application of highly inducible SCOOPs, such as SCOOP2, 3 and 7, could induce IGL levels, which is an interesting aspect that deserves further analysis.

Previous studies have demonstrated a dampened immune response upon on *S. littoralis* attack in the PEP-insensitive Arabidopsis mutant *pepr1pepr2*. Indeed, *S. littoralis* performed better on *pepr1pepr2* and this effect was accompanied by reduced accumulation of JA and JA-Ile in the mutant (Klauser et al., 2015). Moreover, it was shown recently that transcript levels of several members of the *PROSCOOP* gene family are induced in Arabidopsis in response to PEP treatment (Gully et al., 2019). An interesting aspect for further investigations will be thus to study if SCOOPs and PEPs convergently modulate the same signaling pathways to strengthen Arabidopsis immunity against herbivorous insects.

MIK2 was previously reported to be a crucial component of Arabidopsis resistance against the fungal pathogen *Fusarium oxysporum* and is required for elicitation of immune signaling in response to proteinaceous *Fusarium* extracts (Van der Does et al., 2017; Coleman et al., 2021). Intriguingly, *Fusarium* proteomes encode several SCOOP-like sequences and the corresponding synthetic peptides induce immune signaling in Arabidopsis in a MIK2-dependent manner (Hou et al., 2021; Rhodes et al., 2021). Therefore, MIK2 exhibits a unique dual recognition ability by perceiving conserved peptide motifs from endogenous phytocytokines and microbial pathogens. Robust immunity of cruciferous plants against several phytopathogenic fungi, including *F. oxysporum*, relies on functional glucosinolate biosynthesis (Humphry et al., 2010; Liu et al., 2021). Hence, the involvement of SCOOP peptides as potential modulators of glucosinolate biosynthesis to strengthen immunity against fungal pathogens is an additional intriguing aspect for future investigations.

In summary, our data indicate that SCOOP peptide perception by the LLR-RK MIK2 contributes to Arabidopsis resistance against herbivorous insects by promoting JA and IGL biosynthesis. These results illustrate how phytocytokine-mediated signaling modulates a core defense pathway that is initiated by the primary recognition of HAMPs and wounding.

## Data Availability Statement

The raw data supporting the conclusions of this article will be made available by the authors, without undue reservation.

## Author Contributions

ES, AFM, and GG conducted the experiments and evaluated the data under the supervision of PR. M-CG, SA, and J-PR performed initial experiments in a preliminary phase of the project. ES and PR conceptualized the research and wrote the manuscript with feedback from all authors.

## Conflict of Interest

The authors declare that the research was conducted in the absence of any commercial or financial relationships that could be construed as a potential conflict of interest.

## Publisher’s Note

All claims expressed in this article are solely those of the authors and do not necessarily represent those of their affiliated organizations, or those of the publisher, the editors and the reviewers. Any product that may be evaluated in this article, or claim that may be made by its manufacturer, is not guaranteed or endorsed by the publisher.

## Supplementary Material

Supplementary Figure S1 Insect performance of *Spodoptera littoralis* and *Pieris brassicae* on Col-0 and *mik2-1*. **(A)** Representative photographs of Col-0 and *mik2-1* plants (above) and larvae (below) after 12 days of *S. littoralis* feeding. **(B)** Insect performance of *P. brassicae* on Col-0 and *mik2-1*. *P. brassicae* larvae were feeding on 5-week-old plants for 10 days. Means ± SEM of three independent biological replicates are shown. Asterisks denote statistical differences between weights of *P. brassicae* larvae feeding on Col-0 or *mik2-1*: **P* < 0.05 (Mann–Whitney *U* test). Symbols indicate individual values and symbol shapes (circle, square and triangle) indicate different biological replicates.

Supplementary Figure S2 Expression of JA-related genes in Col-0 and *mik2-1*. Expression levels of *MYC2*, *VSP2*, *JAZ5*, and *JAZ10* was measured by qPCR after 2 days of *Spodoptera littoralis* feeding and normalized to the housekeeping gene *SAND*. Non-infested plants served as controls. Values represent means ± SEM of three independent biological replicates. Asterisks denote statistical differences between *S. littoralis-*induced expression levels of Col-0 and *mik2-1*: **P* < 0.05, n.s., no significant difference (ratio paired *t*-test). Different symbols indicate different biological replicates.

Supplementary Figure S3 Depletion of *PROSCOOP12* does not affect Arabidopsis immunity against the specialist *Pieris brassicae*. Insect performance of *P. brassicae* on *proscoop12* mutants in Col-0 and Ws backgrounds. *P. brassicae* larvae were feeding on 5-week-old plants for 10 days. Means ± SEM of three independent biological replicates are shown. n.s., no significant difference in larval weights between *proscoop12* mutant and the corresponding wild-type control (Mann–Whitney *U* test). Symbols indicate individual values and symbol shapes (circle, square, and triangle) indicate different biological replicates.

Supplementary Figure S4 Expression of JA-related genes in *proscoop12* and the corresponding wild-type controls. Expression of *MYC2*, *VSP2*, *JAZ5*, and *JAZ10* was measured by qPCR after 2 days of *S. littoralis* feeding on *proscoop12* mutants in Col-0 **(A)** and Ws **(B)** backgrounds. Expression was normalized to the housekeeping gene *SAND*. Non-infested plants served as controls. Values represent means ± SEM of three independent biological replicates. Asterisks denote statistical differences between *S. littoralis-*induced expression levels of *proscoop12* and the corresponding wild-type control: **P* < 0.05, n.s., no significant difference (ratio paired *t*-test). Different symbols indicate different biological replicates.

Supplementary Figure S5 Exogenous application of SCOOP12. **(A)** Left: Production of reactive oxygen species (ROS), in relative light units (RLU), in leaf disks collected from 4-week-old Col-0 and *mik2-1* plants elicited by 1 μM SCOOP12. Data points represent means from 12 individual leaf disks ± SEM. Right: Integrated ROS production in the same assay over 60 min. Values represent means ± SEM. Letters denote statistical differences (ANOVA followed by Tukey’s HSD). Symbols indicate individual leaf disks. **(B,C)** Glucosinolate levels in Col-0 and *mik2-1* upon SCOOP12 treatment. Absolute levels of total IGLs **(B)** and AGLs **(C)** were measured by UPLC-QTOF 24 h after infiltration with 1 μM SCOOP12. H_2_O-infiltrated plants served as controls. Values represent means ± SEM of three independent biological replicates. Letters denote statistical differences (ANOVA followed by Tukey’s HSD). Different symbols indicate different biological replicates. Values for individual glucosinolate species are given in Supplementary Table S3. **(D)** Expression of *CYP79B2*, *CYP79B3* and *MYC2* was measured by qPCR 24 h after SCOOP12 infiltration (1 μM) and normalized to the housekeeping gene *SAND*. H_2_O-infiltrated plants served as controls. Letters denote statistical differences (ANOVA followed by Tukey’s HSD). Different symbols indicate different biological replicates.
