# Supplementary figures and images for: The MIK2/SCOOP Signaling System Contributes to Arabidopsis Resistance Against Herbivory by Modulating Jasmonate and Indole Glucosinolate Biosynthesis
Source: Front Plant Sci. 2022 Mar 23;13:852808. doi: 10.3389/fpls.2022.852808 (PMC8984487; doi:10.3389/fpls.2022.852808)

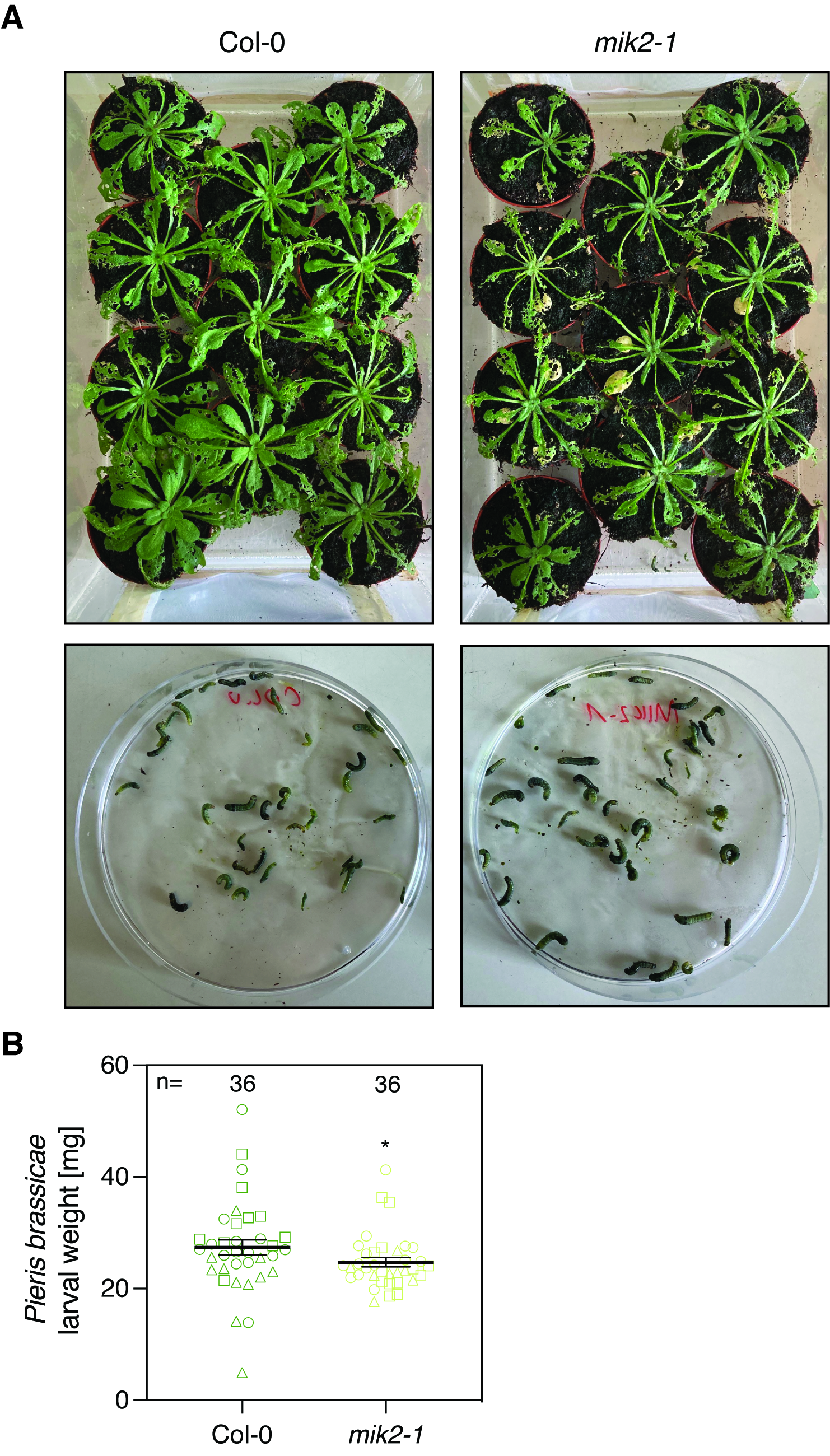

Supplement: Supplementary Figure S1 — Insect performance of Spodoptera littoralis and Pieris brassicae on Col-0 and mik2-1. (A) Representative photographs of Col-0 and mik2-1 plants (above) and larvae (below) after 12 days of S. littoralis feeding. (B) Insect performance of P. brassicae on Col-0 and mik2-1. P. brassicae larvae were feeding on 5-week-old plants for 10 days. Means ± SEM of three independent biological replicates are shown. Asterisks denote statistical differences between weights of P. brassicae larvae feeding on Col-0 or mik2-1: *P < 0.05 (Mann–Whitney U test). Symbols indicate individual values and symbol shapes (circle, square and triangle) indicate different biological replicates. [file Image_1.TIF]

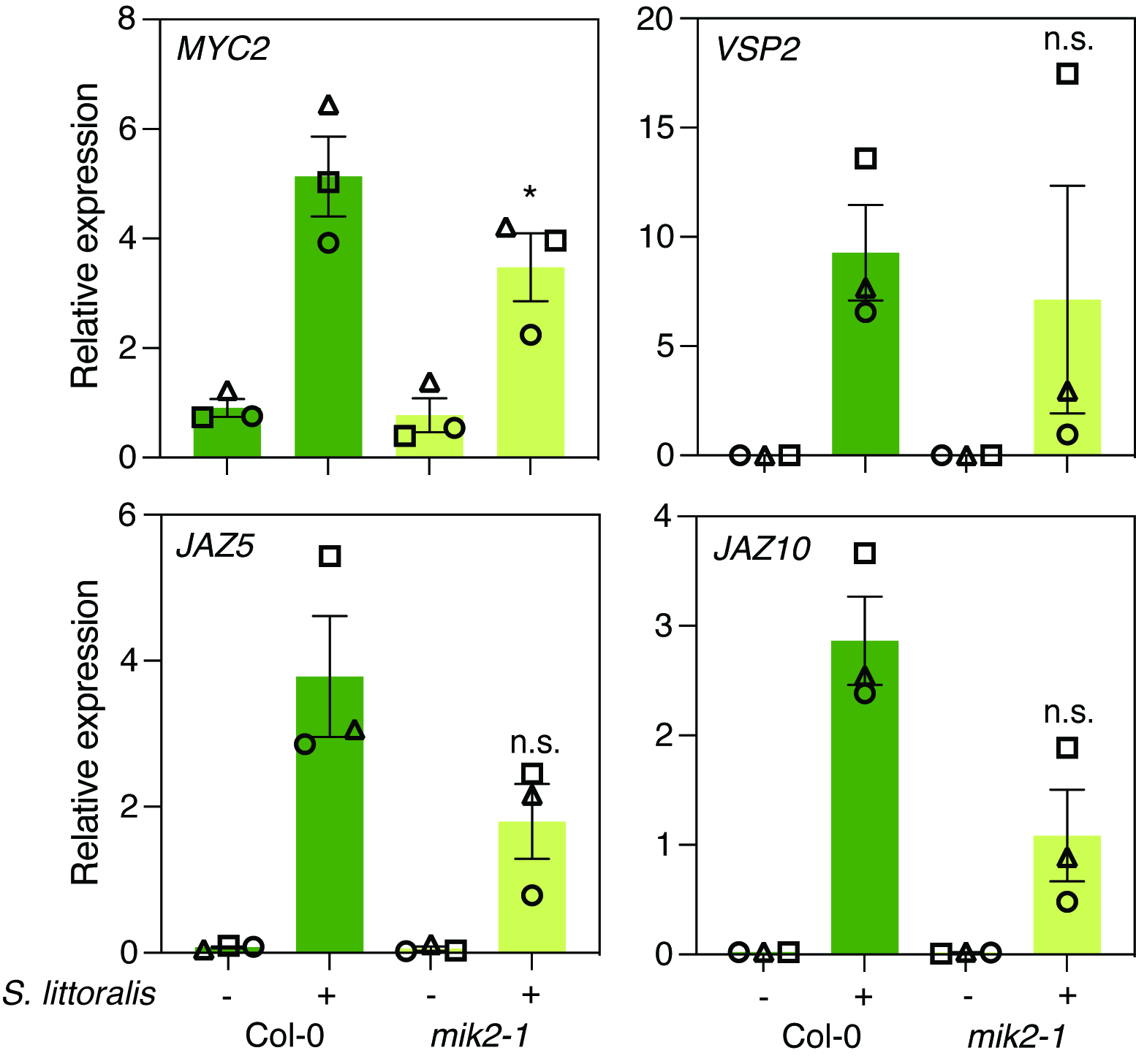

Supplement: Supplementary Figure S2 — Expression of JA-related genes in Col-0 and mik2-1. Expression levels of MYC2, VSP2, JAZ5, and JAZ10 was measured by qPCR after 2 days of Spodoptera littoralis feeding and normalized to the housekeeping gene SAND. Non-infested plants served as controls. Values represent means ± SEM of three independent biological replicates. Asterisks denote statistical differences between S. littoralis-induced expression levels of Col-0 and mik2-1: *P < 0.05, n.s., no significant difference (ratio paired t-test). Different symbols indicate different biological replicates. [file Image_2.TIF]

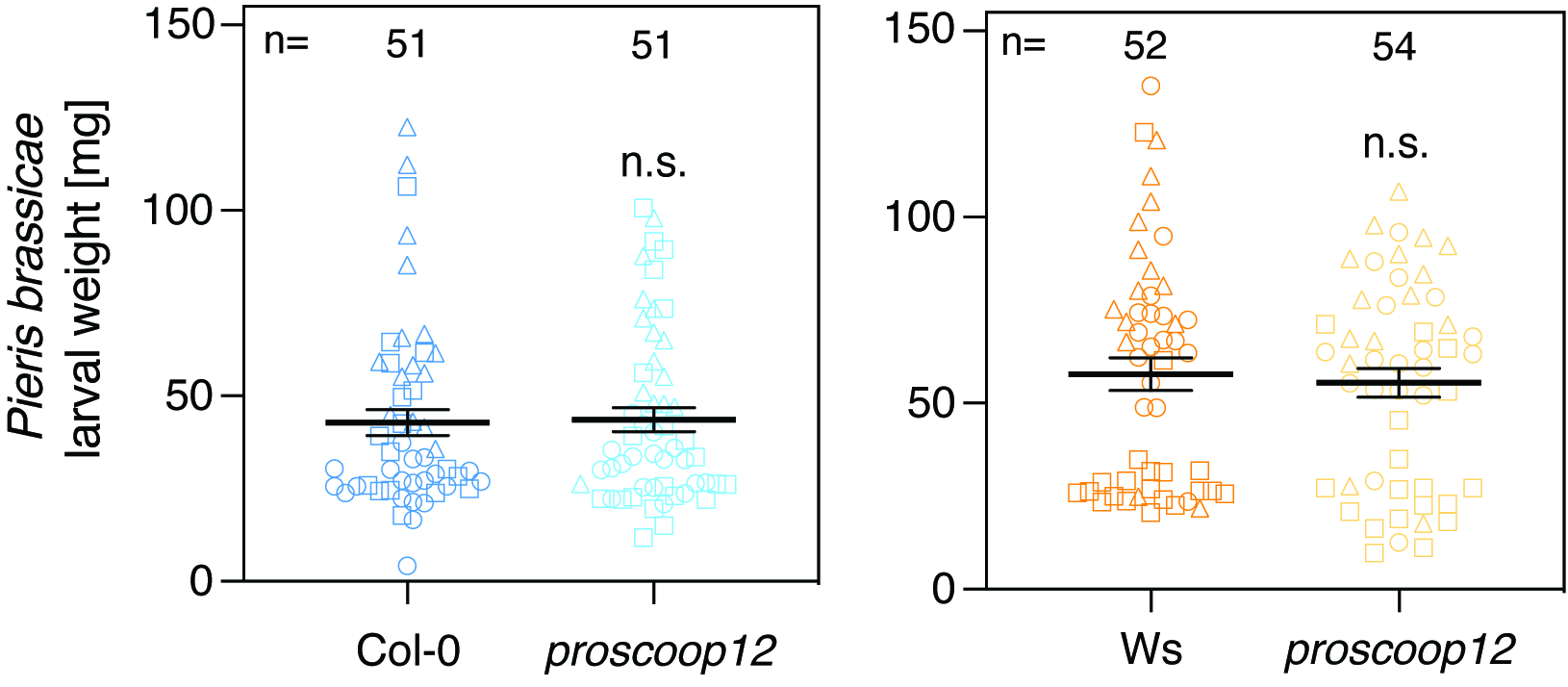

Supplement: Supplementary Figure S3 — Depletion of PROSCOOP12 does not affect Arabidopsis immunity against the specialist Pieris brassicae. Insect performance of P. brassicae on proscoop12 mutants in Col-0 and Ws backgrounds. P. brassicae larvae were feeding on 5-week-old plants for 10 days. Means ± SEM of three independent biological replicates are shown. n.s., no significant difference in larval weights between proscoop12 mutant and the corresponding wild-type control (Mann–Whitney U test). Symbols indicate individual values and symbol shapes (circle, square, and triangle) indicate different biological replicates. [file Image_3.TIF]

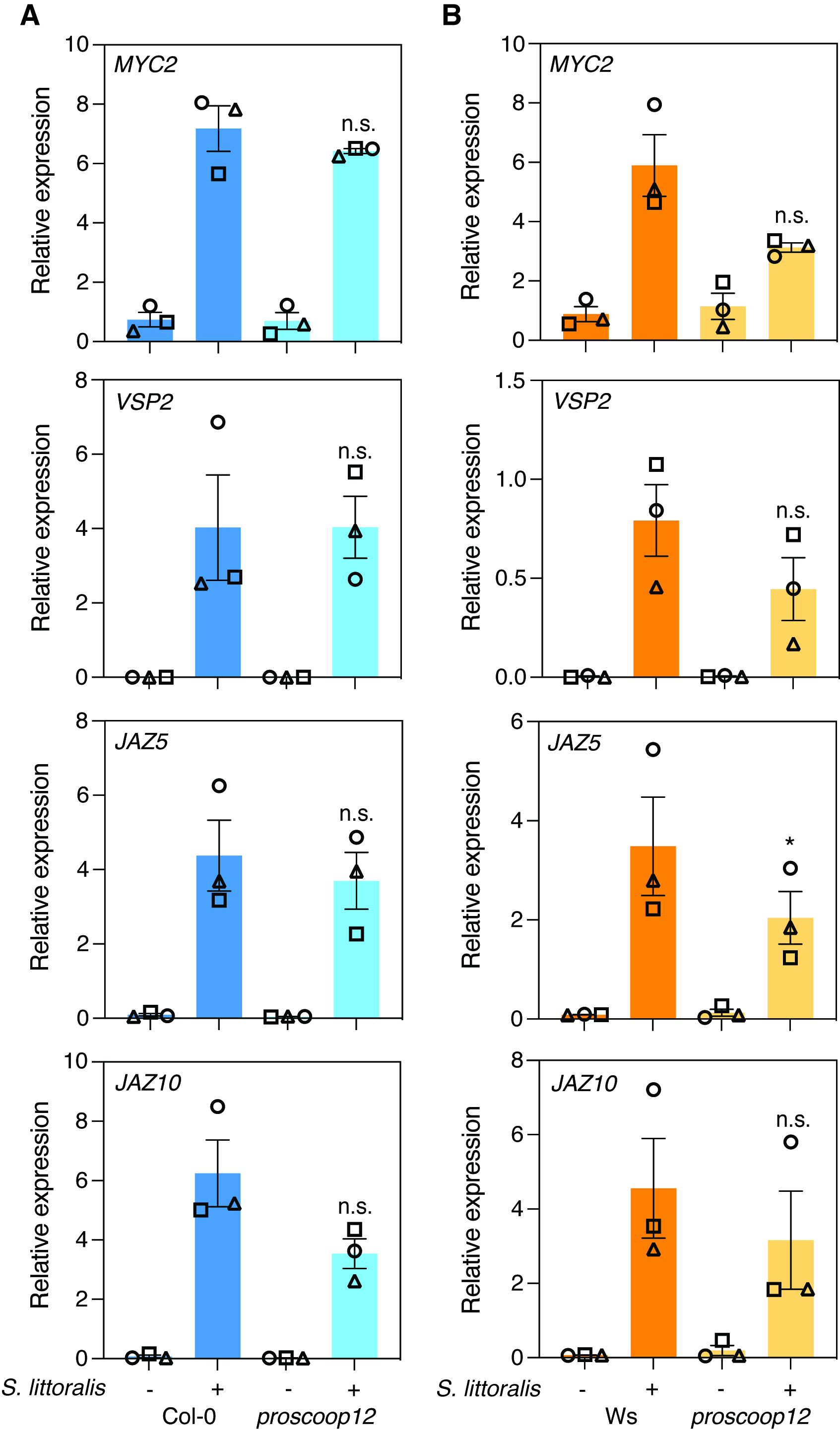

Supplement: Supplementary Figure S4 — Expression of JA-related genes in proscoop12 and the corresponding wild-type controls. Expression of MYC2, VSP2, JAZ5, and JAZ10 was measured by qPCR after 2 days of S. littoralis feeding on proscoop12 mutants in Col-0 (A) and Ws (B) backgrounds. Expression was normalized to the housekeeping gene SAND. Non-infested plants served as controls. Values represent means ± SEM of three independent biological replicates. Asterisks denote statistical differences between S. littoralis-induced expression levels of proscoop12 and the corresponding wild-type control: *P < 0.05, n.s., no significant difference (ratio paired t-test). Different symbols indicate different biological replicates. [file Image_4.TIF]

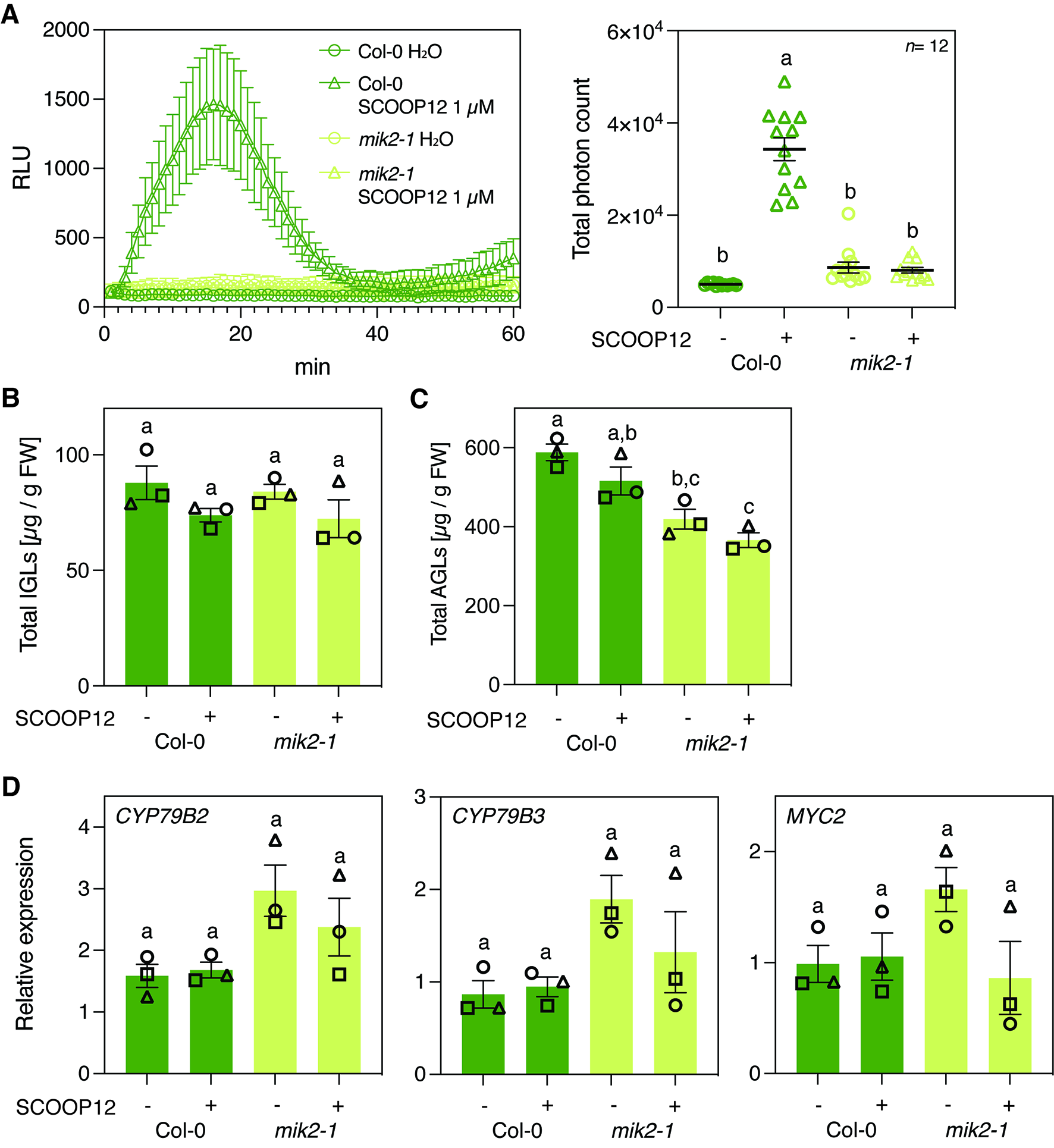

Supplement: Supplementary Figure S5 — Exogenous application of SCOOP12. (A) Left: Production of reactive oxygen species (ROS), in relative light units (RLU), in leaf disks collected from 4-week-old Col-0 and mik2-1 plants elicited by 1 μM SCOOP12. Data points represent means from 12 individual leaf disks ± SEM. Right: Integrated ROS production in the same assay over 60 min. Values represent means ± SEM. Letters denote statistical differences (ANOVA followed by Tukey’s HSD). Symbols indicate individual leaf disks. (B,C) Glucosinolate levels in Col-0 and mik2-1 upon SCOOP12 treatment. Absolute levels of total IGLs (B) and AGLs (C) were measured by UPLC-QTOF 24 h after infiltration with 1 μM SCOOP12. H2O-infiltrated plants served as controls. Values represent means ± SEM of three independent biological replicates. Letters denote statistical differences (ANOVA followed by Tukey’s HSD). Different symbols indicate different biological replicates. Values for individual glucosinolate species are given in Supplementary Table S3. (D) Expression of CYP79B2, CYP79B3 and MYC2 was measured by qPCR 24 h after SCOOP12 infiltration (1 μM) and normalized to the housekeeping gene SAND. H2O-infiltrated plants served as controls. Letters denote statistical differences (ANOVA followed by Tukey’s HSD). Different symbols indicate different biological replicates. [file Image_5.TIF]
