# Supplementary material for: The MIK2/SCOOP Signaling System Contributes to Arabidopsis Resistance Against Herbivory by Modulating Jasmonate and Indole Glucosinolate Biosynthesis
Source: Front Plant Sci. 2022 Mar 23;13:852808. doi: 10.3389/fpls.2022.852808 (PMC8984487; doi:10.3389/fpls.2022.852808)
Supplement: Supplementary file 6 [file Table_1.DOCX]

**Table S1**: Single glucosinolate species in Col-0 and *mik2-1* in response to *Spodoptera littoralis* infestation.

| Metabolite | Abb. | Col-0 | | | | *mik2-1* | | | |
| --- | --- | --- | --- | --- | --- | --- | --- | --- | --- |
|  |  | Ctl | | *S. littoralis* | | Ctl | | *S. littoralis* | |
| Glucoiberin^1^ | 3MSOP | 32.69 | ± 4.73 a | 29.57 | ± 2.56 a | 38.67 | ± 5.74 a | 27.48 | ± 3.43 a |
| Glucoraphanin^1^ | 4MSOB | 242.86 | ± 36.4 a | 243.69 | ± 22.7 a | 308.94 | ± 38.17 a | 245.53 | ± 23.2 a |
| Glucoalyssin^1^ | 5MSOP | 7.17 | ± 0.73 a | 9.23 | ± 0.74 a | 8.33 | ± 0.36 a | 8.37 | ± 0.77 a |
| Glucohesperin^1^ | 6MSOH | 1.03 | ± 0.24 a | 1.38 | ± 0.17 a | 0.79 | ± 0.16 a | 0.76 | ± 0.11 a |
| Glucoibarin^1^ | 7MSOH | 5.11 | ± 1.00 a | 4.92 | ± 0.52 a | 4.62 | ± 0.55 a | 4.04 | ± 0.79 a |
| Glucohirsutin^1^ | 8MSOO | 65.45 | ± 12 a | 37.44 | ± 0.35 a | 62.16 | ± 10.89 a | 37.13 | ± 8.29 a |
| Glucoerucin^1^ | 4MTB | 149.41 | ± 13.5 a | 119.85 | ± 15.1 a | 151.95 | ± 23.69 a | 88.91 | ± 16.8 a |
| Glucoberteroin^1^ | 5MTB | 10.47 | ± 0.91 a | 12.3 | ± 0.75 a | 9.28 | ± 0.7 a,b | 7.11 | ± 0.45 b |
| Gluconasturtiin^1^ | 2PE | 1.69 | ± 0.16 a | 2.03 | ± 0.19 a | 1.79 | ± 0.17 a | 1.66 | ± 0.13 a |
| 7-Methylthioheptyl-GS^1^ | 7MTH | 19.59 | ± 0.70 b | 28.86 | ± 2.73 a | 15.27 | ± 1.27 b | 13.08 | ± 2.23 b |
| 8-Methylthiooctyl-GS^1^ | 8MTO | 80.32 | ± 2.86 a | 79.38 | ± 1.05 a | 72.32 | ± 6.98 b | 44.89 | ± 8.19 b |
| Glucobrassicin^2^ | I3M | 84.35 | ± 7.01 b | 314.64 | ± 21.2 a | 90.51 | ± 7.89 b | 146.57 | ± 15.3 b |
| Hydroxyglucobrassicin^2^ | OH-I3M | 3.29 | ± 0.35 c | 11.93 | ± 0.66 a | 3.4 | ± 0.30 c | 6.48 | ± 0.56 b |
| Methoxyglucobrassicin^2^ | 4MOI3M | 6.2 | ± 0.37 a | 7.66 | ± 0.41 a | 7.08 | ± 0.56 a | 6.35 | ± 0.62 a |
| Neoglucobrassicin^2^ | 1MOI3M | 1.17 | ± 0.20 c | 27.58 | ± 4.73 a | 1.49 | ± 0.06 c | 11.81 | ± 4.4 b,c |

Levels of single aliphatic^1^ and indole^2^ glucosinolate species in Col-0 and *mik2-1* after two days of *S. littoralis* feeding. Non-infested plants served as controls (Ctl). Glucosinolate levels are given in µg g^-1^ FW and represent means ± SEM of three independent biological replicates. Letters denote statistical differences (ANOVA followed by Tukey’s HSD). Total aliphatic and indole glucosinolate are shown in Fig. 1B and C. Abb. = Abbreviation.
