# Supplementary material for: The MIK2/SCOOP Signaling System Contributes to Arabidopsis Resistance Against Herbivory by Modulating Jasmonate and Indole Glucosinolate Biosynthesis
Source: Front Plant Sci. 2022 Mar 23;13:852808. doi: 10.3389/fpls.2022.852808 (PMC8984487; doi:10.3389/fpls.2022.852808)
Supplement: Supplementary file 7 [file Table_2.DOCX]

**Table S2**: Single glucosinolate species in *proscoop12* mutants and corresponding wild-type controls upon *S. littoralis* infestation.

| Metabolite | Abb. | Col-0 | | | | *proscoop12* | | | |
| --- | --- | --- | --- | --- | --- | --- | --- | --- | --- |
|  |  | Ctl | | *S. littoralis* | | Ctl | | *S. littoralis* | |
| Glucoiberin^1^ | 3MSOP | 36.81 | ± 2.80 a | 42.98 | ± 1.95 a | 42.84 | ± 3.52 a | 44.20 | ± 4.34 a |
| Glucoraphanin^1^ | 4MSOB | 236.21 | ± 22.61 a | 294.35 | ± 21.3 a | 282.72 | ± 38.9 a | 309.12 | ± 21.42 a |
| Glucoalyssin^1^ | 5MSOP | 7.79 | ± 0.70 a | 11.58 | ± 0.78 a | 8.23 | ± 1.29 a | 10.67 | ± 0.32 a |
| Glucohesperin^1^ | 6MSOH | 1.19 | ± 0.18 a,b | 1.74 | ± 0.08 a | 0.96 | ± 0.16 b | 1.11 | ± 0.13 a,b |
| Glucoibarin^1^ | 7MSOH | 5.39 | ± 1.04 a | 6 | ± 0.55 a | 4.61 | ± 0.72 a | 4.5 | ± 0.72 a |
| Glucohirsutin^1^ | 8MSOO | 65.49 | ± 12.71 a | 53.75 | ± 4.04 a | 57.24 | ± 8.37 a | 44.3 | ± 10.23 a |
| Glucoerucin^1^ | 4MTB | 162.4 | ± 21.2 a,b | 194.25 | ± 24.5 a | 155.81 | ± 18.6 b | 153.07 | ± 31.2 a,b |
| Glucoberteroin^1^ | 5MTB | 9.73 | ± 0.91 a,b | 12.96 | ± 0.73 a | 8.14 | ± 1.20 b | 9.43 | ± 0.39 a,b |
| Gluconasturtiin^1^ | 2PE | 1.85 | ± 0.21 a | 2.73 | ± 0.18 a | 1.91 | ± 0.24 a | 2.57 | ± 0.15 a |
| 7-Methylthioheptyl-GS^1^ | 7MTH | 20.57 | ± 3.92 a | 26.47 | ± 1.17 a | 15.52 | ± 2.37 a | 16.51 | ± 1.86 a |
| 8-Methylthiooctyl-GS^1^ | 8MTO | 84.08 | ± 15.50 a | 98.92 | ± 10.6 a | 62.35 | ± 10.7 a | 60.27 | ± 60.27 a |
| Glucobrassicin^2^ | I3M | 55.89 | ± 2.18 b | 163.39 | ± 22.4 a | 57.23 | ± 6.14 b | 117.09 | ± 3.78 a |
| Hydroxyglucobrassicin^2^ | OH-I3M | 7.81 | ± 0.21 c | 22.74 | ± 2.50 a | 8.03 | ± 0.49 c | 16.83 | ± 0.33 b |
| Methoxyglucobrassicin ^2^ | 4MOI3M | 14.06 | ± 3.36 a | 22.9 | ± 2.68 a | 13.24 | ± 2.57 a | 16.9 | ± 1.86 a |
| Neoglucobrassicin^2^ | 1MOI3M | 2.9 | ± 0.26 a | 28.89 | ± 14.6 a | 4.01 | ± 0.96 a | 11.97 | ± 1.32 a |
|  |  | Ws | | | | *proscoop12* | | | |
|  |  | Ctl | | *S. littoralis* | | Ctl | | *S. littoralis* | |
| Glucoiberin^1^ | 3MSOP | 563.56 | ± 73.72 a | 657.46 | ± 77.5 a | 415.09 | ± 61.33a | 552.11 | ± 112.3 a |
| Glucoraphanin^1^ | 4MSOB | 6.17 | ± 0.82 a | 8.68 | ± 0.34 a | 5.40 | ± 0.65 a | 7.74 | ± 0.99 a |
| Glucoalyssin^1^ | 5MSOP | 0.24 | ± 0.03 a | 0.38 | ± 0.04 a | 0.26 | ± 0.03 a | 0.36 | ± 0.03 a |
| Glucohesperin^1^ | 6MSOH | 0.22 | ± 0.06 a | 0.43 | ± 0.02 a | 0.22 | ± 0.03 a | 0.39 | ± 0.09 a |
| Glucoibarin^1^ | 7MSOH | 2.18 | ± 0.10 a | 2.54 | ± 0.12 a | 2.06 | ± 0.43 a | 2.52 | ± 0.59 a |
| Glucohirsutin^1^ | 8MSOO | 70.67 | ± 6.82 a | 65.50 | ± 5.21 a | 55.16 | ± 14.3 a | 58.72 | ± 17.77 a |
| Glucoerucin^1^ | 4MTB | 2.34 | ± 0.08 a | 3.83 | ± 0.20 a | 1.56 | ± 0.55 a | 2.53 | ± 0.76 a |
| Glucoberteroin^1^ | 5MTB | 0.35 | ± 0.04 a | 0.47 | ± 0.02 a | 0.32 | ± 0.03 a | 0.42 | ± 0.03 a |
| Gluconasturtiin^1^ | 2PE | 0.07 | ± 0.003 a | 0.08 | ± 0.01 a | 0.08 | ± 0.05 a | 0.05 | ± 0.03 a |
| 7-Methylthioheptyl-GS^1^ | 7MTH | 8.12 | ± 0.69 b | 12.23 | ± 0.58 a | 7.16 | ± 0.77 b | 10.03 | ± 0.73 a,b |
| 8-Methylthiooctyl-GS^1^ | 8MTO | 94.01 | ± 12.9 a,b | 136.45 | ± 9.16 a | 68.23 | ± 9.29 b | 96.58 | ± 11.1 a,b |
| Glucobrassicin^2^ | I3M | 110.08 | ± 9.91 c | 212.56 | ± 11.8 a | 108.44 | ± 2.05 c | 153.96 | ± 5.26 b |
| Hydroxyglucobrassicin^2^ | OH-I3M | 14.38 | ± 1.15 b | 29.67 | ± 2.70 a | 14.78 | ± 1.13 b | 21.85 | ± 1.01 b |
| Methoxyglucobrassicin^2^ | 4MOI3M | 8.61 | ± 1.84 b | 15.98 | ± 1.24 a | 7.04 | ± 0.59 b | 12.67 | ± 2.02 a,b |
| Neoglucobrassicin^2^ | 1MOI3M | 1.62 | ± 0.09 a | 1.25 | ± 0.1a,b | 1.51 | ± 0.1 a,b | 1.11 | ± 0.05 b |

Levels of single aliphatic^1^ and indole^2^ glucosinolate species in *proscoop12* mutants and corresponding wild-type controls after two days of *S. littoralis* feeding. Non-infested plants served as controls (Ctl). Glucosinolate levels are given in µg g^-1^ FW and represent means ± SEM of three independent biological replicates. Letters denote statistical differences (ANOVA followed by Tukey’s HSD). Total aliphatic and indole glucosinolate are shown in Fig. 3B and C. Abb. = Abbreviation.
