# Supplementary material for: The MIK2/SCOOP Signaling System Contributes to Arabidopsis Resistance Against Herbivory by Modulating Jasmonate and Indole Glucosinolate Biosynthesis
Source: Front Plant Sci. 2022 Mar 23;13:852808. doi: 10.3389/fpls.2022.852808 (PMC8984487; doi:10.3389/fpls.2022.852808)
Supplement: Supplementary file 8 [file Table_3.DOCX]

**Table S3**: Single glucosinolate species in Col-0 and *mik2-1* upon SCOOP12 treatment.

| Metabolite | Abb. | Col-0 | | | | *mik2-1* | | | |
| --- | --- | --- | --- | --- | --- | --- | --- | --- | --- |
|  |  | H_2_O | | SCOOP12 | | H_2_O | | SCOOP12 | |
| Glucoiberin^1^ | 3MSOP | 30.01 | ± 0.73 a | 26.39 | ± 1.04 a | 24.68 | ± 1.36 a | 21.28 | ± 1.09 b |
| Glucoraphanin^1^ | 4MSOB | 225.17 | ± 1.84 a | 197.15 | ± 12.1 a | 197.58 | ± 14.27 a | 175.23 | ± 14.3 a |
| Glucoalyssin^1^ | 5MSOP | 7.09 | ± 0.25 a | 6.27 | ± 0.31 a | 6.31 | ± 0.46 a | 5.91 | ± 0.46 a |
| Glucohesperin^1^ | 6MSOH | 0.9 | ± 0.06 a | 0.78 | ± 0.1 a | 0.34 | ± 0.03 b | 0.36 | ± 0.03 b |
| Glucoibarin^1^ | 7MSOH | 4.18 | ± 0.24 a | 3.57 | ± 0.37 a | 2.03 | ± 0.18 b | 1.87 | ± 0.15 b |
| Glucohirsutin^1^ | 8MSOO | 55.8 | ± 3.03 a | 47.1 | ± 5.94 a | 21.8 | ± 2.27 b | 20.13 | ± 1.91 b |
| Glucoerucin^1^ | 4MTB | 157.6 | ± 13.9 a | 133.4 | ± 9 a,b | 102.2 | ± 5 b,c | 80.6 | ± 3.13 c |
| Glucoberteroin^1^ | 5MTB | 11.2 | ± 0.51 a | 10.39 | ± 0.46 a | 8.1 | ± 0.3 b | 7.21 | ± 0.41 b |
| Gluconasturtiin^1^ | 2PE | 1.65 | ± 0.01 a | 1.35 | ± 0.1 a,b | 1.14 | ± 0.1 b,c | 1.01 | ± 0.08 c |
| 7-Methylthioheptyl-GS^1^ | 7MTH | 17.8 | ± 0.47 a | 17.75 | ± 0.5 a | 10.91 | ± 0.45 b | 10.26 | ± 0.51 b |
| 8-Methylthiooctyl-GS^1^ | 8MTO | 76.56 | ± 3.41 a | 71.53 | ± 5.54 a | 43.75 | ± 2.15 b | 42.22 | ± 3.06 b |
| Glucobrassicin^2^ | I3M | 78.69 | ± 6.61 a | 65 | ± 2.14 a | 75.26 | ± 2.86 a | 63.91 | ± 7.71 a |
| Hydroxyglucobrassicin^2^ | OH-I3M | 3.71 | ± 0.5 a | 2.8 | ± 0.2 a,b | 2.82 | ± 0.1 a,b | 2.26 | ± 0.17 b |
| Methoxyglucobrassicin^2^ | 4MOI3M | 4.26 | ± 0.1 a | 4.82 | ± 0.24 a | 4.84 | ± 0.2 a | 5.1 | ± 0.32 a |
| Neoglucobrassicin^2^ | 1MOI3M | 1.21 | ± 0.22 a | 1.21 | ± 0.41 a | 1.1 | ± 0.17 a | 1.1 | ± 0.05 a |

Levels of single aliphatic^1^ and indole^2^ glucosinolate species in Col-0 and *mik2-1* infiltrated with 1 µM SCOOP12 for 24 h. H_2_O-infiltrated plants served as controls. Glucosinolate levels are given in µg g^-1^ FW and represent means ± SEM of three independent biological replicates. Letters denote statistical differences (ANOVA followed by Tukey’s HSD). Total aliphatic and indole glucosinolate are shown in Fig. S5B and C. Abb. = Abbreviation.
