# Supplementary material for: The MIK2/SCOOP Signaling System Contributes to Arabidopsis Resistance Against Herbivory by Modulating Jasmonate and Indole Glucosinolate Biosynthesis
Source: Front Plant Sci. 2022 Mar 23;13:852808. doi: 10.3389/fpls.2022.852808 (PMC8984487; doi:10.3389/fpls.2022.852808)
Supplement: Supplementary file 9 [file Table_4.DOCX]

**Table S4**: Relative expression of *PROSCOOP1* to *PROSCOOP14* in Col-0 and Ws after two days of *S. littorralis* feeding.

|  | Col-0 | | | | Ratio |  |
| --- | --- | --- | --- | --- | --- | --- |
|  | Ctl | | *S. littoralis* | | *S.l.*/Ctl |  |
| *PROSCOOP1* | 0.05 | ± 0.016 | 0.11 | ± 0.02 | 2.19 |  |
| *PROSCOOP2* | 0.01 | ± 0.002 | 0.28 | ± 0.08 | 21.83 | ** |
| *PROSCOOP3* | 2E-05 | ± 3.9E-06 | 0.0016 | ± 7.4E-05 | 61.16 |  |
| *PROSCOOP4* | 1.64 | ± 0.42 | 1.59 | ± 0.58 | 0.97 |  |
| *PROSCOOP5* | 0.0008 | ± 6.1E-05 | 0.0073 | ± 1.2E-03 | 9.51 | * |
| *PROSCOOP6* | 0.08 | ± 0.01 | 1.03 | ± 0.12 | 12.97 | ** |
| *PROSCOOP7* | 0.005 | ± 0.0004 | 0.19 | ± 0.03 | 35.10 | *** |
| *PROSCOOP8* | 0.09 | ± 0.01 | 1.29 | ± 0.18 | 14.72 | ** |
| *PROSCOOP9* | 0.24 | ± 0.05 | 0.19 | ± 0.06 | 0.81 |  |
| *PROSCOOP10* | 24.15 | ± 3.32 | 31.57 | ± 6.31 | 1.31 |  |
| *PROSCOOP11* | 0.18 | ± 0.03 | 0.16 | ± 0.01 | 0.85 |  |
| *PROSCOOP12* | 0.05 | ± 0.01 | 0.17 | ± 0.11 | 3.72 |  |
| *PROSCOOP13* | 0.08 | ± 0.02 | 0.07 | ± 0.01 | 0.9 |  |
| *PROSCOOP14* | 2.3 | ± 1.01 | 2.71 | ± 0.57 | 1.17 |  |
|  | Ws | | | | Ratio |  |
|  | Ctl | | *S. littoralis* | | *S.l.*/Ctl |  |
| *PROSCOOP1* | 0.003 | ± 0.001 | 0.01 | ± 0.002 | 3.72 |  |
| *PROSCOOP2* | 0.01 | ± 0.003 | 0.75 | ± 0.31 | 54.19 | * |
| *PROSCOOP3* | 0.0001 | ± 3.8E-05 | 0.0002 | ± 3.8E-05 | 1.34 |  |
| *PROSCOOP4* | 0.21 | ± 0.12 | 0.59 | ± 0.04 | 2.80 |  |
| *PROSCOOP5* | 0.0002 | ± 6.9E-05 | 0.0002 | ± 5.1E-05 | 0.97 |  |
| *PROSCOOP6* | n.d. |  | n.d. |  |  |  |
| *PROSCOOP7* | 0.003 | ± 0.0017 | 0.015 | ± 0.006 | 4.89 |  |
| *PROSCOOP8* | 0.77 | ± 0.49 | 1.59 | ± 0.16 | 2.06 |  |
| *PROSCOOP9* | 0.13 | ± 0.06 | 0.14 | ± 0.04 | 1.08 |  |
| *PROSCOOP10* | 39.84 | ± 14.21 | 64.69 | ± 21.61 | 1.62 |  |
| *PROSCOOP11* | 0.07 | ± 0.01 | 0.05 | ± 0.01 | 0.71 |  |
| *PROSCOOP12* | 0.05 | ± 0.03 | 0.13 | ± 0.07 | 2.74 | * |
| *PROSCOOP13* | 0.05 | ± 0.01 | 0.05 | ± 0.001 | 0.89 |  |
| *PROSCOOP14* | 0.94 | ± 0.53 | 0.92 | ± 0.03 | 0.99 |  |

Expression of *PROSCOOP1* to *PROSCOOP14* relative to the housekeeping gene *SAND* was measured by qPCR after two days of *S. littoralis* feeding. Non-infested plants served as controls (Ctl). Ratios for single PROSCOOPs normalized to the expression levels in non-infested plants are shown in Fig. 5A. Values represent means ± SEM of three independent biological replicates. Asterisks denote statistical differences between *S. littoralis*- and non-infested plants: **P* < 0.05, ***P* < 0.01, ****P* < 0.001 (Ratio paired *t*-test). n.d. = not detectable.
