# Supplementary material for: The MIK2/SCOOP Signaling System Contributes to Arabidopsis Resistance Against Herbivory by Modulating Jasmonate and Indole Glucosinolate Biosynthesis
Source: Front Plant Sci. 2022 Mar 23;13:852808. doi: 10.3389/fpls.2022.852808 (PMC8984487; doi:10.3389/fpls.2022.852808)
Supplement: Supplementary file 10 [file Table_5.DOCX]

**Table S5**: Relative expression of *PROSCOOP1* to *PROSCOOP14* in Col-0 upon mechanical wounding.

|  |  | | | | |  | | Ratio |  | |  | | Ratio |
| --- | --- | --- | --- | --- | --- | --- | --- | --- | --- | --- | --- | --- | --- |
|  | Ctl | |  | 4 hpw | |  | | W/Ctl | 24 hpw | |  | | W/Ctl |
| *PROSCOOP1* | 0.05 | ± 0.02 |  | 0.11 | ± 0.03 |  | | 2.14 | 0.01 | ± 0.005 | |  | 0.22 |
| *PROSCOOP2* | 0.01 | ± 0.005 |  | 0.02 | ± 0.004 |  | | 1.47 | 0.04 | ± 0.008 | | * | 1.47 |
| *PROSCOOP3* | 9E-05 | ± 0.00004 |  | 2E-04 | ± 0.0001 | |  | 2.04 | 5E-04 | ± 0.0003 | |  | 5.75 |
| *PROSCOOP4* | 0.59 | ± 0.29 |  | 3.11 | ± 0.68 | |  | 5.26 | 0.85 | ± 0.36 | |  | 1.44 |
| *PROSCOOP5* | 0.004 | ± 0.002 |  | 0.13 | ± 0.08 | |  | 34.6 | 0.01 | ± 0.003 | |  | 2.61 |
| *PROSCOOP6* | 0.09 | ± 0.02 |  | 0.92 | ± 0.23 | * | | 10.34 | 0.72 | ± 0.30 | | * | 8.08 |
| *PROSCOOP7* | 0.002 | ± 0.0005 |  | 0.12 | ± 0.04 | * | | 57.96 | 0.06 | ± 0.01 | * | | 31.12 |
| *PROSCOOP8* | 0.06 | ± 0.04 |  | 1.47 | ± 0.68 | * | | 22.73 | 0.49 | ± 0.13 |  | | 7.51 |
| *PROSCOOP9* | 0.07 | ± 0.01 |  | 0.09 | ± 0.01 |  | | 1.28 | 0.03 | ± 0.01 |  | | 0.48 |
| *PROSCOOP10* | 10.8 | ± 1.33 |  | 18.11 | ± 1.81 |  | | 1.67 | 12.85 | ± 1.63 |  | | 1.18 |
| *PROSCOOP11* | 0.13 | ± 0.01 |  | 0.09 | ± 0.02 |  | | 0.71 | 0.05 | ± 0.01 |  | | 0.42 |
| *PROSCOOP12* | 0.05 | ± 0.01 |  | 0.11 | ± 0.03 |  | | 1.99 | 0.05 | ± 0.01 | * | | 0.86 |
| *PROSCOOP13* | 0.07 | ± 0.02 |  | 0.06 | ± 0.01 |  | | 0.87 | 0.07 | ± 0.03 |  | | 1.00 |
| *PROSCOOP14* | 1.31 | ± 0.50 |  | 1.59 | ± 0.44 |  | | 1.22 | 3.1 | ± 1.05 |  | | 2.37 |

Expression of *PROSCOOP1* to *PROSCOOP14* relative to the housekeeping gene *SAND* was measured by qPCR 4 and 24 hours after mechanical wounding. Non-wounded plants served as controls (Ctl). Ratios for single PROSCOOPs normalized to the expression levels in non-wounded plants are shown in Fig. 5B. Values represent means ± SEM of three independent biological replicates. Asterisks denote statistical differences between wounded and non-wounded plants at different time-points: **P* < 0.05 (Ratio paired *t*-test). hpw = hours post wounding.
