# Supplementary material for: The MIK2/SCOOP Signaling System Contributes to Arabidopsis Resistance Against Herbivory by Modulating Jasmonate and Indole Glucosinolate Biosynthesis
Source: Front Plant Sci. 2022 Mar 23;13:852808. doi: 10.3389/fpls.2022.852808 (PMC8984487; doi:10.3389/fpls.2022.852808)
Supplement: Supplementary file 11 [file Table_6.DOCX]

**Table S6:** List of primers used for qPCR analysis in this study.

| Gene | AGI Code | Forward | Reverse |
| --- | --- | --- | --- |
| *SAND* | At2g28390 | AACTCTATGCAGCATTTGATCCACT | TGATTGCATATCTTTATCGCCATC |
| *CYP79B2* | At4G39950 | CCCACCATTAAGGAGCTTGT | CTCCGGTTTGTTCACCATCT |
| *CYP79B3* | At2G22330 | CTTTGCTTACCGCTGATGAA | GCGTTTGATGGGTTGTCTG |
| *CYP83B1* | At4G31500 | CCGCCCTAAACAAGAAACAG | GGCCTTGACATTTTCGTGAG |
| *GSTF9* | At2G30860 | TCGCTCTACAGCCTTTTGGT | CCCAAAAGATCAGGTCCTTG |
| *MYC2* | At1G32640 | GTGCGGGATTAGCTGGTAAA | ATGCATCCCAAACACTCCTC |
| *VSP2* | At5G24770 | GGTTGATGCTCCGGTCCCTAACCA | GGTGCCCGCAAATTGCAAAGACTA |
| *JAZ5* | At1G17380 | ATTCATTCCTGCCATCTTGC | AGCAGGAACAGCTTCAAACC |
| *JAZ10* | At5G13220 | TACTATCCGACATCGGCCTAA | GTATACGATTTAGCAACGACG |
| *PROSCOOP1* | At5G44565 | AGCATCCTCTTTCACCATACCG | ATTCTGACCACCACCACCTC |
| *PROSCOOP2* | At5G44567 | TAATTGTGCTGGTCTCATGCTC | GCGGTGGCGGCGGTTTTT |
| *PROSCOOP3* | At5G00585 | GGTCCTTTGAATTTGAGACTTTTG | TAATACGAGCTCTTCGACCATAC |
| *PROSCOOP4* | At5G44568 | ATCTCAAGTTGGAGTCGCCC | TTATCTTTAGGCGATGCAGAGTGA |
| *PROSCOOP5* | At5G44570 | ATACAATCCACCGACGCTGC | GGATAGAGCATTTGTGGCTGC |
| *PROSCOOP6* | At5G44572 | CTTGCAGCCTTAGCCAATCG | TCATCAATCTCCTCCCGTGG |
| *PROSCOOP7* | At5G44574 | CACTTGCCTTAGCGTAACGG | TGGTGAGTTTTCTCCACGCT |
| *PROSCOOP8* | At5G44575 | TCCCAACCCATACGGAGTCT | TTTGTTGACCACCACCGGC |
| *PROSCOOP9* | At5G44578 | TCCGTATCCGTATGGTGGCA | ATGCTGCTACCACCATGTCC |
| *PROSCOOP10* | At5G44580 | TGGGGAGGAAGCGGATGAAT | CACTGCCTGATGGTCCTGTA |
| *PROSCOOP11* | At5G44582 | TTGTAATCACTGGAAGGAGG | GTTGCGACCACCACCTTGT |
| *PROSCOOP12* | At5G44585 | TTCTTCTCCTCTGCACCGTC | TAAAACGTCCACCAGCTTGG |
| *PROSCOOP13* | At1G22885 | TGATATCCTTTCAAGTTGGAGTCG | TTATGGACCTTTTCCTTTGCGC |
| *PROSCOOP14* | At1G22890 | CTCACAAGTTGGACTAGGCGA | GGGCCTTGTCCTTTGTGTGA |
